# Deep contrastive learning for predicting cancer prognosis using gene expression values

**DOI:** 10.1093/bib/bbae544

**Published:** 2024-10-29

**Authors:** Anchen Sun, Elizabeth J Franzmann, Zhibin Chen, Xiaodong Cai

**Affiliations:** Department of Electrical and Computer Engineering, University of Miami, Miami, FL 33146, United States; Department of Otolaryngology, University of Miami, Miami, FL 33146, United States; Sylvester Comprehensive Cancer Center, University of Miami, Miami, FL 33146, United States; Sylvester Comprehensive Cancer Center, University of Miami, Miami, FL 33146, United States; Department of Microbiology and Immunology, University of Miami, Miami, FL 33146, United States; Department of Electrical and Computer Engineering, University of Miami, Miami, FL 33146, United States; Sylvester Comprehensive Cancer Center, University of Miami, Miami, FL 33146, United States

**Keywords:** cancer prognosis, gene expression, machine learning, contrastive learning, survival analysis

## Abstract

Recent advancements in image classification have demonstrated that contrastive learning (CL) can aid in further learning tasks by acquiring good feature representation from a limited number of data samples. In this paper, we applied CL to tumor transcriptomes and clinical data to learn feature representations in a low-dimensional space. We then utilized these learned features to train a classifier to categorize tumors into a high- or low-risk group of recurrence. Using data from The Cancer Genome Atlas (TCGA), we demonstrated that CL can significantly improve classification accuracy. Specifically, our CL-based classifiers achieved an area under the receiver operating characteristic curve (AUC) greater than 0.8 for 14 types of cancer, and an AUC greater than 0.9 for 3 types of cancer. We also developed CL-based Cox (CLCox) models for predicting cancer prognosis. Our CLCox models trained with the TCGA data outperformed existing methods significantly in predicting the prognosis of 19 types of cancer under consideration. The performance of CLCox models and CL-based classifiers trained with TCGA lung and prostate cancer data were validated using the data from two independent cohorts. We also show that the CLCox model trained with the whole transcriptome significantly outperforms the Cox model trained with the 16 genes of Oncotype DX that is in clinical use for breast cancer patients. The trained models and the Python codes are publicly accessible and provide a valuable resource that will potentially find clinical applications for many types of cancer.

## Introduction

Numerous gene prognostic signatures have been developed over the last two decades for various types of cancer such as breast cancer [1, 2], colorectal cancer [3, 4], lung cancer [5, 6], and prostate cancer [7]. These signatures use the expression values of several to several hundred selected genes to predict a cancer patient’s prognosis. While gene prognostic signatures are crucial in determining personalized treatment, only a few of them have proven values in clinical trials, and are currently used in practice. These include Oncotype DX [8] and MammaPrint [9, 10] for breast cancer. Oncotype DX uses the expression values of 21 genes to calculate a cancer recurrence score (RS), while MammaPrint uses the expression values of 70 genes to classify patients into a high- or low-risk group of recurrence. A critical step of developing these gene signatures was selecting a set of genes that can provide adequate predictive power for cancer prognosis, which remains challenging.

To avoid the step of gene selection, the Cox regression model has been employed to predict prognosis and outcome of cancer based on expression values of a large number of genes [11, 12]. Efficient algorithms have been developed to infer such high-dimensional Cox regression model by maximizing the partial likelihood regularized by the ridge (Cox-ridge), Lasso (Cox-Lasso), or elastic net (Cox-EN) penalty [11]. More recently, artificial neural networks (ANNs) in conjunction with the Cox model have been employed to predict cancer prognosis [13–19]. SurvivalNet [13], DeepSurv [14], and Cox-nnet [15] used a fully connected ANN or multilayer perceptron (MLP). They provided comparable performance with Cox-ridge or Cox-EN in most types of cancer; SruvivalNet and Cox-nnet offered slightly better performance than Cox-ridge or Cox-EN in one out of three cancers and in two out of ten cancers, respectively.

The performance of ANNs [13–15] was limited possibly by the relatively small number of data samples of each cancer type available to train the model. One approach to mitigating this problem is to employ a method that can exploit data across multiple types of cancer. Towards this end, the meta-learning method [20] and the variational autoencoder [21] have been adopted in prediction of cancer prognosis [18, 19]. The meta-learning method achieved comparable or slightly better performance than direct training in lung cancer, glioma, and headneck squamous cell carcinoma [18], while the performance of VAECox in terms of the estimated concordance index (c-index) was slightly better than that of Cox-nnet in 7 out of 10 caners, but it is not clear if difference of the performance reaches any statistical significance.

Contrastive learning (CL) uses a deep ANN to effectively learn feature representations from unlabeled and/or labeled data, which can be used to aid in other learning tasks [22]. In this study, supervised CL [23] is applied to predict cancer prognosis using gene expression values. A classifier is first developed, utilizing features learned by a CL-based ANN to categorize patients into low- or high-risk groups for recurrence. This classification approach is similar to the one used by MammaPrint [9, 10]. A CL-based Cox model (CLCox) is then developed, in which CL is used to learn feature representations that are input to the Cox model for predicting the hazard ratio (HR), which can serve as a prognostic index [24], similar to the RS of Oncotype DX [8], to stratify patients into different risk groups for personalized treatment. Using datasets from The Cancer Genome Atlas (TCGA) [25], it is demonstrated that CL can significantly improve prediction accuracy. Furthermore, CL-based classifiers and CLCox models trained with the TCGA data for lung and prostate cancer are validated by utilizing data from two independent cohorts. Finally, it is shown that the CLCox model trained with expression values of all 13,235 genes in a breast cancer dataset offers significantly better performance than the model trained with expression values of 16 genes of Oncotype DX.

## Results

### Overview of machine learning models

**Figure 1:**
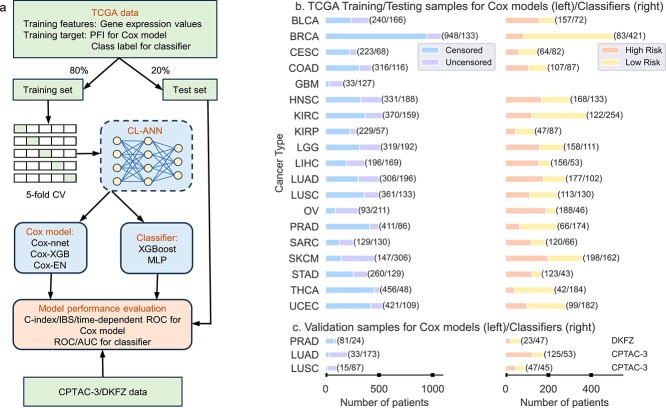
Overview of development of machine learning models for predicting cancer prognosis. (a) Flow chart for training and testing Cox models and classifiers. For each of 19 types of cancer, TCGA gene expression and clinical data were randomly split into a training set (80% samples) and a test set (20% samples). The training data were used to train a contrastive learning-based artificial neural network (CL-ANN). The features learned by CL-ANN were used to train a Cox model to predict HR or a classifier to categorize patients into a high or low risk group of disease recurrence. Five-fold CV was employed to determine optimal hyperparameters of the model. Test data were then used to evaluate the performance of the trained model. Data from CPTAC-3 and DKFZ were further used to validate the models of LUAD, LUSC, and PRAD trained with TCGA data. This process of random splitting of data, training, testing, and validating each model was repeated 40 times. (b) List of 19 types of cancer and the numbers of TCGA samples for training and testing Cox models (left) and classifiers (right). (c) Numbers of samples of lung cancer (LUAD and LUSC) and prostate cancer (PRAD) from two independent cohorts, CPTAC-3 and DKFZ, for validating the Cox models (left) and classifiers (right) trained with TCGA data.

As depicted in Fig. 1(a), our CL-based machine learning models consist of two modules: a CL module to learn feature representations from tumor transcriptomes and a Cox proportional hazards model or a classifier to predict cancer prognosis using the learned features from the CL model. The CL module is an MLP trained with a contrastive loss function [23]. The Cox model was implemented using three existing methods: (1) Cox-EN [11], (2) Cox-XGB that is the gradient boosting-based approach implemented with XGBoost [26], and (3) Cox-nnet that is an ANN-based model [15]. The Cox model was trained using features from the CL module and progress free intervals (PFIs) of the patients. We referred the three CL-based models to as CLCox-EN, CLCox-XGB, and CLCox-nnet, respectively. The Cox model predicts HR that can be used as a prognostic index [24] similar to the RS of Oncotype DX [8]. We also used the classification approach, as used by MammaPrint [9, 10], to classify patients into a high- or low-risk group of cancer recurrence. The gradient boosting classifier implemented with XGBoost [26] and MLP were adopted. Patients were divided into two groups: a high risk group with a PFI $< 3 $ years and a low risk group with a PFI $\ge $ 3 years, and the classifier was trained using features from the CL module and class labels. We referred the CL-based classifiers as CL-XGBoost and CL-MLP. For performance comparison, we also bypassed the CL module and trained the Cox model and the classifier directly using the gene expression values.

We selected the TCGA datasets of 19 types of cancer, and trained and tested machine learning models for each type of cancer. The names of these 19 cancer types and their abbreviations are given in the Datasets section of the Supplemental Text. Figure 1(b) shows the number of patients of each type of cancer. Of note, the number of samples for classification is smaller than that for training the Cox model, because the samples with an censored PFI $<3$ years do not have a class label and cannot be used in classification. GBM was not used in classification because the number of samples was too small. For each type of cancer, we used 80% of randomly selected data samples to train the CL model, the Cox model and the classifier, and the remaining 20% data to evaluate the performance of the overall model. Five-fold cross validation (CV) was used to selected the optimal values of hyperparameters of each model and the architecture of the ANN during training. For performance evaluation, we used the receiver operating characteristic (ROC) curve and the area under the ROC curve (AUC) for the classifier, and Harrell’s c-index, the integrated Brier score (IBS), and the time-dependent ROC curve for the Cox model. The process of random splitting data into a training set and a test set, training and testing the model was repeated 40 times. This process is called Monte Carlo CV [27, 28]. Of note, the test data were never used to train the CL model, the classifier, or the Cox model.

While the models trained with the TCGA training data were evaluated with the TCGA test data that were not used in training, we also used data of two independent cohorts to validate the models trained with TCGA data. Specifically, the lung cancer data of the Clinical Proteomic Tumor Analysis Consortium 3 (CPTAC-3) [29] and a prostate cancer dataset named DKFZ [30] were used to validate models trained with TCGA LUAD, LUSC, and PRAD data, respectively. The numbers of samples in the CPTAC-3 and DKFZ datasets are given in Fig. 1(c).

### Contrastive learning improves risk prediction

We trained a classifier to categorize patients into a high- or low-risk group of cancer recurrence, as done by MammaPrint [9, 10]. The architecture of the MLP in the CL module determined by five-fold CV had two hidden layers for all 18 types of cancer. The number of nodes at each hidden layer and the output layer varied for different types of cancer. Specifically, the number of nodes is 5,396 or 5,196 at the first hidden layer, 2,048 or 1,024 at the second hidden layer, and 256 or 128 at the output layer. The feature at the output of the MLP was chosen to train a XGBoost or MLP classifier. The MLP classifier had two hidden layers, and the number of nodes at the first and second layers is in the following sets: {(2,048, 64), (1,024, 32), (512, 32), (512, 16)}. To see the effect of CL, we also bypassed the CL module in Fig. 1(a) and trained a XGBoost or MLP classifier using the gene expression values as input.

**Figure 2:**
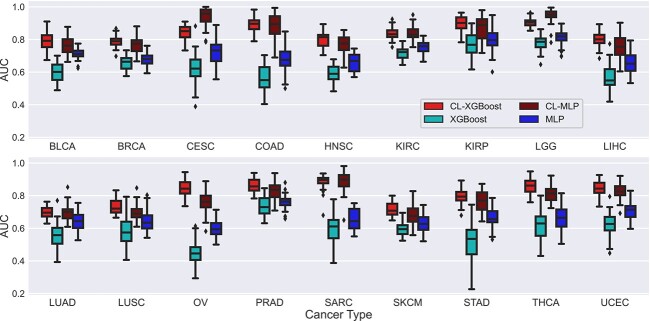
Performance of the XGBoost and MLP classifiers with and without contrastive learning for classifying cancer patients into a high- or low-risk group of disease recurrence. Two risk groups were defined by a cut-off value of three progression free years.

Figure 2 presents the AUCs of the four classifiers with or without CL for 18 types of cancer; Figs S1 and S2 show the corresponding ROC curves. It is observed that CL significantly improved the AUC of XGBoost by more than 0.1 for all 18 types of cancer with a $P$-value less than 0.05 (Wilcoxon rank-sum test). CL increased the AUC of XGBoost by more than 0.2 for 9 types of cancer (CESC, COAD, HNSC, LIHC, OV, SARC, STAD, THCA, and UCEC) and by more than 0.3 for 2 types of cancer (COAD and OV). The AUC of CL-XGBoost exceeds 0.8 for 12 types of cancer, whereas the AUC of XGBoost is less than 0.8 for all 18 types of cancer. Notably, the AUC of CL-XGBoost is 0.906 for LGG and 0.896 for KIRP. CL significantly improved the AUC of MLP by more than 0.05 for all 18 types of cancer, by more than 0.1 for 10 types of cancer (CESC, COAD, HNSC, LGG, LIHC, OV, SARC, STAD, THCA, UCEC), and by more than 0.2 for 3 types of cancer (CESC, COAD, and SARC). The AUC of CL-MLP exceeds 0.8 for 8 types of cancer (CESC, COAD, KIRC, KIRP, LGG, SARC, THCA, UCEC), whereas the AUC of MLP is less than 0.8 for 17 types of cancer except LGG. Remarkably, the AUC of CL-MLP is 0.945 for LGG and 0.943 for CESC. The AUC of CL-XGBoost is higher than or almost the same as that of CL-MLP in most types of cancer except CESC and LGG.

Results in Fig. 2 were obtained by defining two classes with a cut-off values of three progression free years. Since different types of cancer may generally have different prognosis, we may need different cut-off values for different types of cancer. Therefore, based on the distribution of PFIs, we used a cut-off value of 5 years of PFI to define two classes for BRCA and KIRC. Similarly, we used a cut-off value of 2 years to define two classes for LUAD and LUSC. Figure S3 shows the AUCs of the classifiers for these four types of cancer under the two cut-off values mentioned. CL increased the average AUC by 0.211, 0.137, 0.252, and 0.067 for BRCA, KIRC, LUAD, and LUSC, respectively.

### Data pooling improves risk prediction

The clustering analysis of TCGA RNA-seq data by Hoadley *et al.* [31] revealed that gene expression profiles of certain types of cancer were in the same cluster. We reasoned that if the data in the same cluster or group are pooled to train a model, we might improve prediction accuracy especially for those types of cancer with a relatively small number of samples. Towards this end, we identified eight groups of cancers reported by Hoadley *et al.* [31]. As described in the Supplemental Text, we used pooled data to train a CL-based XGBoost classifier, which is referred to as CLg-XGBoost, for each of 12 types of cancer that belongs to at least one group.

**Figure 3:**
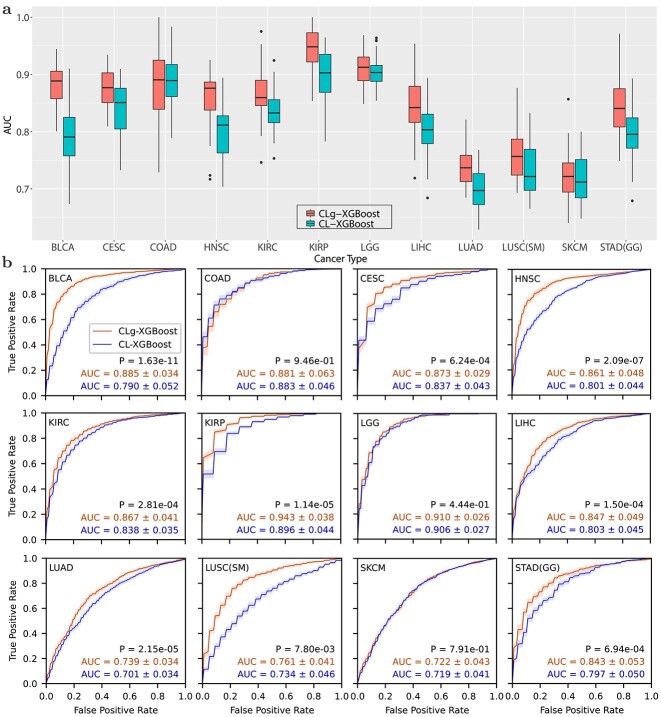
Performance of CL-based classifiers trained with the data of a single type of cancer and the pooled data of a group of different types of cancer. (a) Box plots of AUCs for 12 types of cancer, each of which belongs to at least one of the 9 groups. CLg-XGBoost represents the CL-XGBoost classifier trained with the pooled data. LUSC (SM) and STAD (GG) standard for the LUSC and STAD classifiers trained with CL features learned from the data in the squamous morphology (SM) and the gastrointestinal group (GG), respectively. (b) ROCs of CLg-XGBoost and CL-XGBoost for 12 types of cancer. Two risk groups were defined by a cut-off value of three progression free years.

Figure 3 compares the ROCs and AUCs obtained using the pooled data from a group of cancer types and the data of individual type of cancer. Data pooling achieved statistically significant improvement for 9 out of 12 types of cancer except the following three types of cancer: LGG, COAD, and SKCM. In particular, the AUC for BLCA and STAD was improved from 0.790 to 0.885, and from 0.797 to 0.843, respectively. Remarkably, CLg-XGBoost for KIRP and LGG achieved an AUC of 0.943 and 0.910, respectively. Results in Figs 2 and 3 show that CL boosted the AUC of the XGBoost classifier above 0.8 for 14 out of 18 types of cancer.

### Contrastive learning improves prediction of the HR

We compared the performance of six methods that used the Cox model to predict the HR of 19 types of cancer using the TCGA data. These six methods include three existing methods, Cox-nnet, Cox-EN, and Cox-XGB, and the three methods, CLCox-nnet, CLCox-EN, and CLCox-XGB, that we developed to combine CL with the three existing methods. The final MLP in the CL module determined by five-fold CV had two hidden layers. The number of nodes at each layer might be different for different types of cancer. Generally, the first and second hidden layers and the output layer had 5,196 − 5,596, 3,096 − 4,096, and 1,024 − 2,048 nodes, respectively. The features at the output of the first hidden layer were selected to train a Cox model. Five-fold CV was also used to determine the hyper-parameters of the Cox model. In particular, the ANN of Cox-nnet had one hidden layer as also used in [15].

**Figure 4:**
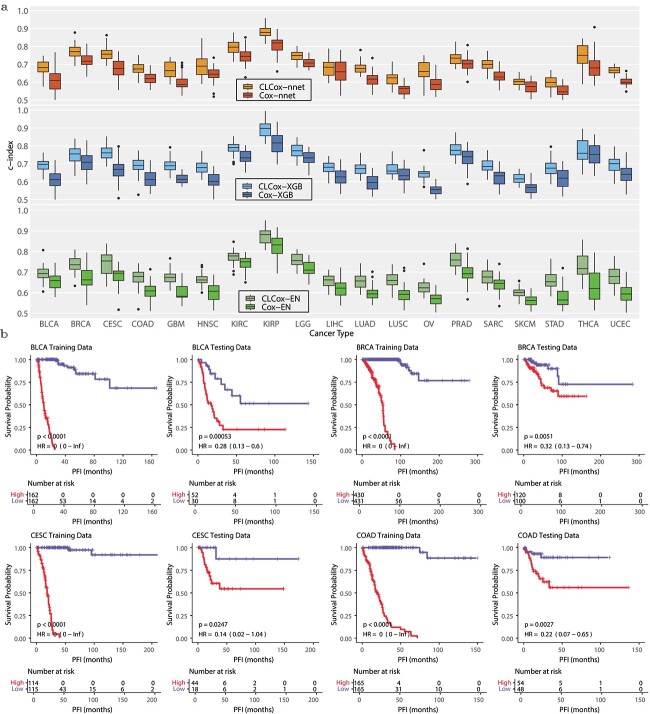
Performance of Cox models in predicting the HR of 19 types of cancer using the TCGA data. (a) Box-plots of c-indexes of Cox models. (b) KM-curves for the two groups of patients stratified by the median HR predicted by the CLCox-XGB model using the training data: a poorly prognostic group (red) with patients’ HR greater than the median HR and a better prognostic group (blue) with patients’ HR less than the median HR. The KM-curves for the remaining 15 types of cancer are presented in Figs S8 and S9.

Figure 4(a) shows the box plots of c-indexes of the six Cox models trained with TCGA data. It is seen that CL improved the prediction for all 19 types of cancer. To see the performance improvement more clearly, Table S1 lists the c-indexes and their standard errors, as well as $P$-values (Wilcoxon rank-sum test) for the comparison of two methods with and without CL. The increase of the c-index produced by CL is statistically significant ($P$-value $< 0.05$) for 55 out of 57 comparisons except the following two comparisons: CLCox-nnet versus Cox-nnet for LIHC and CLCox-XGB versus Cox-XGB for THCA. Averaging across all types of cancer, CL increased the c-index by 0.058, 0.062, and 0.060 for Cox-nnet, Cox-XGB, and Cox-EN, respectively. The largest c-index is 0.880 achieved by CLCox-EN for KIRP. Figure S4 shows the IBS of the six Cox models. It is observed that each CLCOx model achieved a smaller or almost the same IBS for every type of cancer comparing with the model without CL. Figures S5- S7 depict the time-dependent ROC curves of Cox-EN and CLCox-EN. CLCox-EN provides a significant better AUC for all 19 types of cancer at all four time points considered.

To see if the HR predicted by a Cox model can distinguish patients with different prognosis, we stratified the patients of a type of cancer into two groups using the median HR in the training set. Figure 4(b) depicts the Kaplan–Meier (KM) curves of these two groups of cancer patients in the training and test sets of four types of cancer. The KM curves of the remaining 15 other types of cancer are shown in Figs S8 and S9. The KM curves of the two groups of cancer patients are statistically different ($P$-value $< 0.05$, log-rank test) for every type of cancer. This shows the HRs predicted by our models are able to discriminate patients with different prognosis.

### Data pooling improves prediction of HR

As described earlier, we considered eight groups of cancer with similar patterns of gene expression and used the pooled data within a group to train a classifier, which resulted in improved classification accuracy. We also used the pooled data to train Cox models aiming to improve performance. Three Cox models trained with pooled data are referred to as CLgCox-EN, CLgCox-XGB, and CLgCox-nnet. Figure S10 compares the c-indexes of CL models trained with the data of a single cancer or pooled data of multiple types of cancer in a group, and Table S2 lists the values of these c-indexes and the $P$-values (Wilcoxon sum-rank test) for the comparisons. Data pooling provided better or similar c-indexes for almost all 13 types of cancer that belong to at least one group and three Cox models, except for the following five comparisons: CLgCox-nnet vs. CLCox-nnet for HNSC, CLgCox-XGB vs. CLCox-XGB for CESC, HNSC, and LUSC(SM), and CLgCox-EN vs. CLCox-EN for KIRP. Performance improvement is statistically significant ($P$-value $<0.05$) in 20 out of 45 comparisons. In particular, data pooling improved the c-index by more than 0.03 for the following three types of cancer LGG, LUSC, and STAD with at least one Cox model. Moreover, CLgCox models with data pooling achieved a lower or almost the same IBS comparing with CLCox models without data pooling as shown in Fig. S10.

### Validation on the independent cohorts

**Figure 5:**
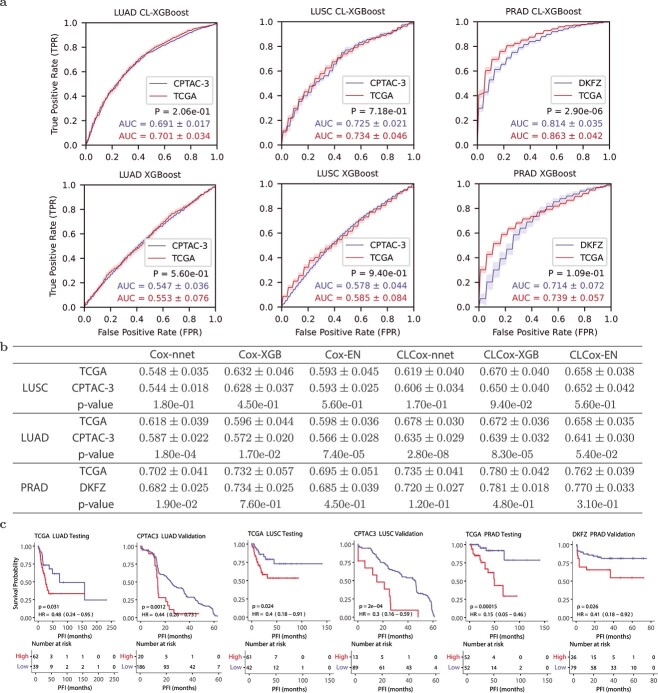
Validation of Cox models and classifiers trained with the TCGA data using two independent cohorts (CPTAC-3 and DKFZ). (a) ROC curves of CL-XGBoost and XGBoost. (b) C-indexes of six Cox models. (c) KM curves for the two groups stratified by the median HR predicted by the CLCox-EN model from the TCGA training data: a high-risk group (red) with HRs > the median HR and a low-risk group (blue) with HRs < the median HR.

We used two independent datasets, the CPTAC-3 lung cancer dataset [29] and the DKFZ prostate cancer dataset [30], to validate classifiers and Cox models trained with the TCGA LUSC, LUAD, and PRAD data, as described in the Supplemental Text. Figure 5(a) compares the two ROCs of each classifier for each of the three types of cancer: one obtained with the TCGA test data and the other obtained with CPTAC-3 or DKFZ data. There is no significant difference in five out of six pairs of AUCs except for that of CL-XGBoost on the PRAD dataset. Although the $P$-value of CL-XGBoost on the PRAD dataset is 2.90e-06, difference between the two AUCs (0.863 and 0.814) is relatively small. This demonstrates that the performance of the classifiers on independent cohorts is largely not different from that on the original TCGA cohort.

Figure 5(b) gives the c-indexes of Cox models obtained with TCGA test data and CPTAC-3 or DKFZ data. Regarding LUSC, c-indexes of each of six Cox models do not exhibit any statistically significant difference for CPTAC-3 and TCGA data. As for LUAD, the CLCox-EN model yielded c-indexes that are not statistically different for CPTAC-3 and TCGA data, while the remaining five models gave statistically different c-indexes on the two datasets. However, the difference of c-indexes between CPTAC-3 and TCGA is relatively small, ranging from 0.017 to 0.043. With respect to the results of PRAD, the $P$-value for the comparison between the c-indexes of Cox-nnet for TCGA and DKFZ data is 0.019, and that the $P$-values for the remaining five methods are all greater than 0.05. This indicates that all six Cox models essentially did not produce statistically different c-indexes on TCGA and DKFZ PRAD data. Figure 5(c) shows the KM curves for the two groups of patients of each of three cancer types, stratified by the median HR predicted by the CLCox-EN model from the TCGA training data. It is observed that the two groups in CPTAC-3 and DKFZ cohorts exhibit statistically different KM curves, similar to that seen in the TCGA test data. Of note, the slight difference in AUCs and c-indexes between the TCGA cohort and the two independent cohorts may be due to the data quality rather than the classifiers, as will be elaborated in Discussion.

### The CLCox model with all genes outperforms the model with 16 genes of Oncotype DX

Oncotype DX uses expression values of 16 genes, which are normalized relative to the expression values a set of 5 housekeeping genes, to compute an RS to predict the risk of distant recurrence of estrogen receptor-positive (ER+) breast cancer patients [8, 32]. Since deep learning can learn features effectively for a specific prediction task [33], we investigated if a CL-based Cox model with expression values of all available genes could offer better performance than the Cox model with the 16 genes of Oncotype DX. As described in the Supplemental Text, the investigation was conducted using a collection of six datasets of breast cancer [34, 35] that contain microarray expression data of 13,235 genes and distant metastasis free survival (DMFS) time of 687 ER+ patients.

**Figure 6:**
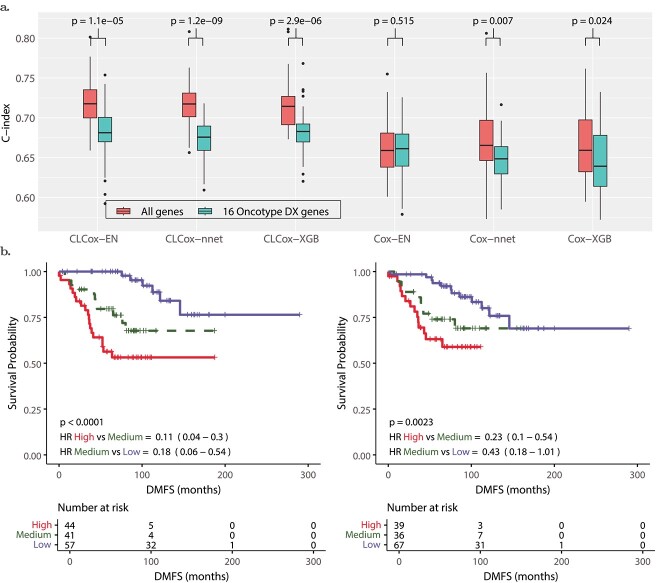
Performance of Cox models in predicting the distant recurrence risk of breast cancer. (a) Box-plots of c-indexes of Cox models. (b) KM-curves for the three groups of patients stratified by the HR predicted by the CLCox-EN model with all 13,235 genes (left) and with 16 genes of Oncotype DX (right): a high-risk group (red) with patients’ HR greater than the 73th percentile of all HRs, a low-risk group prognostic group (blue) with patients’ HR less than the 51st percentile of all HRs, and a medium-risk group with patients’ HR in between the 51st percentile and the 73th percentile.

Figure 6(a) shows that Cox-EN, Cox-XGB, and Cox-nnet models with the 16 genes of Oncotype DX offer a c-index around 0.65, which is similar to the c-index of the univariate Cox model that uses the RS value of the Oncotype DX as the predictor [35]. The c-index of the Cox-EN model with all 13,235 genes is not statistically different from that of the Cox-EN model with the Oncotype DX genes, whereas the c-indexes of Cox-nnet and Cox-XGB with all genes are slightly higher than those of Cox-nnet and Cox-XGB with Oncotype DX genes with a $P$-value less than 0.05.

We then trained CLCox-EN, CLCox-XGB, and CLCox-nnet models with 16 genes of Oncotype DX and also with all 13,235 genes. As shown in Fig. 6(a), CL improves the c-index for all six models. CLCox-EN, CLCox-XGB, and CLCox-nnet models with all genes offer the best c-index of about 0.72, significantly better than those (about 0.68) of CLCox-EN, CLCox-XGB, and CLCox-nnet models with 16 genes of Oncotype DX, and also those (about 0.65) of the six models without CL. Figure 6(b) depicts the KM curves of three risk groups in the test data that were determined using the HRs predicted by the CLCox-EN model in the training data as described in the Supplemental Text. These KM curves show that three groups of patients have statistically different DMFS probabilities. The CLCox-EN model with all genes exhibits a lower $P$-value and lower HRs in pair-wise comparisons (high-risk group vs. medium-risk group and medium-risk group vs. low-risk group) than the CLCox-EN model with 16 genes of Oncotype DX, which corroborates its better prediction accuracy as shown in c-indexes in Fig. 6(a).

## Discussion

CL is a self-supervised machine learning technique typically carried out with data augmentation that uses the structure of the data such as images to generate similar data instances [22]. Supervised CL has also been developed to take class label information into account, which results in an embedding space where elements of the same class are more closely aligned than those in different classes [23]. Gene expression data do not have the structure of images that can facilitate data augmentation. Therefore, we cannot apply self-supervised CL here, but instead use supervised CL. Since we partitioned data samples into multiple groups with similar PFIs, the low-dimensional features learned by the CL method are closely aligned for those data samples in the same group and more separated for those samples in different groups. The Cox models that we developed to rely on these kind of features were able to offer significantly better performance than existing methods, as observed in our results.

The HR output from the Cox model is a continuous number, similar to the RS of Oncotype DX [8], indicating the risk of cancer recurrence. Two cutoff values of RS were determined in a clinical trial [8] to divide breast cancer patients into three risk groups based on their RSs. To stratify patients of a specific cancer type into different risk groups using the HR predicted by our Cox model, clinical trials may be needed to determine cutoff values. As an alternative to the Cox model, we also trained a classifier to directly categorize patients in different risk groups of recurrence, as done by MammaPrint [9, 10]. We demonstrated that CL could significantly improve the accuracy of the classifier. Specifically, for the 18 types of cancer considered, our CL-based classifiers achieved an AUC of between 0.7 and 0.8 for 4 types of cancer, between 0.8 and 0.9 for 11 types of cancer, and above 0.9 for 3 types of cancer, whereas the AUC of the classifier without using CL was below 0.8 for 17 types of cancer. The AUC is an indication of the discriminating power of a classifier. The general guidelines of using AUC to determine the discriminating power are as follows [36, 37]: acceptable discrimination ($0.7\le \text{AUC} < 0.8$), excellent discrimination ($0.8\le \text{AUC} < 0.9$ ), and outstanding discrimination ($\text{AUC} \ge 0.9$). Thus, our CL-based classifiers achieved excellent or outstanding AUC for the vast majority of the 18 types of cancer that we analyzed.

We used data of two independent cohorts named CPTAC-3 and DKFZ to validate the Cox models and classifiers trained with the TCGA data of three types of cancer, LUSC, LUAD, and PRAD. Most c-indexes of the Cox models and AUCs of the classifier do not exhibit statistically significant difference on the TCGA data and the CPTAC-3/DKFZ data. For those c-indexes and AUCs that show statistically significant difference, the differences are relatively small. These differences may be due to the data rather than the methods that trained the models. First, we converted the RPKM values in CPTAC-3 and DKFZ RNA-seq data to read counts per gene used in the TCGA data. If the length of a gene used in data conversion is different from the one used in the TCGA data, the converted value may not be consistent with the value in the TCGA data. Second, 534, 561, and 1215 genes in TCGA LUAD, LUSC, and PRAD data were missing in CPTAC-3 LUAD, CPTAC-3 LUSC, and DKFZ data, respectively. We set the expression values of those missing genes to zero in the validation process. Third, as shown in Fig. 5(c), the maximum PFIs in CPTAC-3 and DKFZ datasets are much shorter than those in TCGA data. Despite these factors that might have affected the validation results, the data of independent cohorts largely validate the models trained with TCGA data, demonstrating that our models are robust.

Oncotype DX uses a set of 16 cancer-related genes and 5 reference genes to compute an RS. These 16 genes were carefully selected from 250 candidate genes reported in the literature using the gene expression and clinical data of 447 breast cancer patients [8]. We demonstrated that our CLCox model trained with all 13,235 genes available in a breast cancer dataset provided significantly better performance than the Cox model trained with 16 genes of Oncotype DX. This indicates that the CL-based ANN can learn better features than manually selected 16 genes of Oncotype DX.

While it is challenging to interpret deep learning models, we may use a gradient-based method such as SmoothGrad [38] or layerwise relevance propagation [39] to identify features that make significant contributions to the output of the model. In our case, we may apply these methods to determine genes that may significantly influence cancer prognosis. However, the primary objective of this paper is to design and train machine learning models that can offer significant predictive capability for cancer prognosis. The prediction of such models can assist in designing personalized treatment [1–7]. Therefore, we did not attempt to interpret the models because of space constraints.

The CL-based classifier for each of 18 types of cancer and the CLCox model for each of 19 types of cancer trained with the TCGA RNA-seq data are available as mentioned in the Data and Code Availability section. If the RNA-seq transcriptome of a new tumor of one of these types of cancer is available, one can normalize the RNA-seq data using the 10 housekeeping genes as described in the Supplemental Text, and then input the normalized data to the corresponding model to predict the recurrence risk of the patient. Python codes for model training are also available, and one can use or modify the codes to train a new CL-based classifier or a CLCox model to possibly improve the prediction of the model, if more data are available. While we used PFI as the clinical endpoint as recommended by Liu *et. al.* [40], one may use a different endpoint such as distant recurrence as used by Oncotype DX [8] and MammaPrint [9, 10] for breast cancer, and retrain classifiers or CLCox models. One may also use a cut-off value different from three progression free years to define risk groups for a specific type of cancer, and retrain the classifiers using our code. In short, the trained models and the Python codes in this study provide a valuable resource that will potentially find clinical applications for many types of cancer.

## Methods

### Datasets

The RNA-seq and clinical data of 19 types of different cancer from TCGA [25] were used to train and test models. RNA-seq and clinical data of lung cancer from CPTAC-3 [29] and a prostate cancer dataset named DKFZ [30] were used to validate the models trained with the TCGA data. A collection of six breast cancer microarray datasets [34, 35] was used to train and test Cox models that use the expression values of 16 genes of Oncotype DX [8] or all 13,325 genes in the dataset as the input. The availability of these datasets is described in the section of Data and Code Availability. More detailed description of these datasets is given in the Supplemental Text, and the number of patients for each type of cancer is given in Fig. 1(b)&(c).

### CL-based classifier

Our CL-based classifier consists of two modules: a CL module that uses an ANN to learn a representation of the high dimensional gene expression vector of a patient in a low-dimensional space, and a classification module that uses the features from the CL module to classify patients into a low- or high-risk of recurrence. The CL module is an MLP with sigmoid activation at hidden layers, and we used the supervised contrastive loss function [23] in training. Suppose that the training data set contains $n$ patients of a particular type of cancer. Let the vector ${\mathbf x}_{i}$, $i=1,2,\cdots , n$, represent the expression values of genes in patient $i$, and $t_{i}$ be the PFI of patient $i$. Without loss of generality, let us assume $t_{1}\le t_{2}\le \cdots \le t_{n}$. We divided $n$ samples into $m$ groups, each of which contains $\lfloor n/m \rfloor $ or $\lfloor n/m \rfloor +1$ samples, where $\lfloor n/m \rfloor $ stands for the largest integer that is less than or equal to $n/m$. We chose $m$ such that $\lfloor n/m \rfloor $ was around 15. For each ${\mathbf x}_{i}$, we obtained a group label $y_{i}$ to be the index of the group that it belongs to.

During the training, we took a mini-batch of $b$ samples $\{{\mathbf x}_{i_{j}}, y_{i_{j}}, j=1,2,\cdots , b\}$, where $\{i_{1},i_{2},\cdots ,$$i_{b}\}$ is a subset of $\{1,2,\cdots ,n\}$. Let us define the set $J:=\{1,2,\cdots , b\}$. If we randomly select a $j\in J$, let $A(j){:=} J\backslash \{j\}$ be the set that contains the elements of $J$ excluding $j$. For a sample $({\mathbf x}_{i_{j}}, y_{i_{j}})$ in the mini-batch, we define a positive set that contains all the samples with the same label as ${\mathbf x}_{i_{j}}$: $P(j):=\{p\in A(j): y_{i_{p}}= y_{i_{j}} \}$. Let ${\mathbf z}_{j}=\mathrm{MLP}({\mathbf x}_{i_{j}})$ be the output the neural network given the input ${\mathbf x}_{i_{j}}$. Then, we used the following contrastive loss function in training the neural network [23]:


\begin{align*} & {{\mathcal{L}}}=\sum_{j\in J}{{\mathcal{L}}}_{j}=\sum_{j \in J}\frac{-1}{|P(j)|} \sum_{p\in P(j)} \log\frac{\exp({\mathbf z}_{j}^{T}{\mathbf z}_{p}/\tau)}{\sum_{a\in A(j)}\exp({\mathbf z}_{j}^{T}{\mathbf z}_{a}/\tau)}, \end{align*}

where $|P(j)|$ is the cardinality of the set $P(j)$, and $\tau $ is a positive constant set to 0.07 in training [23].

We randomly split the dataset of each type of cancer into a training set (80%) and a test set (20%). The training set was used to train the CL module. More specifically, the training data were used to train MLPs with the contrastive loss function. MLPs were built in PyTorch and trained using the SGD optimizer. To mitigate the possible over-fitting problem, we used $\ell _{2}$-regularization and early stopping. One eighth of the training data was set aside as the validation set, and the remaining data were used to train the model. During training, the average loss on the validation data over each epoch was calculated. The minimum validation loss was kept or updated at the end of each epoch. After the number of epoch reaches 50, training can stop at any epoch when the following conditions are met. Starting from the epoch when the minimum validation loss is updated, if the validation loss in an epoch is greater than an threshold equal to the minimum validation loss plus 0.01, or if the validation loss is less than the threshold for 1500 consecutive epochs but without dropping below the minimum validation loss, then training stops. The maximum number of epochs was set to 5000. The hyperparameters include the following: the number of layers (2,3,4,5), the number of nodes at each layer (1024–8192), the learning rate ($10^{-4}, 5\times 10^{-5}, 2\times 10^{-5}, 10^{-5}, 10^{-6}$), and the weight for the $\ell _{2}$-regularization term ($0.01, 0.005, 0.003, 0.001, 0.0007, 0.0003$, and $0.0001$). Five-fold CV was employed to search over the space of the hyperparameters, and five MLPs with the smallest validation loss were selected. Training samples (${\mathbf x}_{i}$s) were input to each of MLP, and the output of each MLP at a hidden layer or the output layer (${\mathbf z}_{i}$s) were used to train a classifier to categorize patients into a low- or high-risk group of recurrence.

To train the classifier, we divided all patients into two groups: a high-risk group with PFI < 3 years and a low-risk group with PFI > 3 years, and labeled the available data samples correspondingly. Of note, those samples that were censored and whose PFI was < 3 years could not be labeled, and therefore they were removed from the data. For all the samples in the training set, we got their outputs from the CL model and used them to train a gradient boosting classifier, which was implemented with XGBoost [26], or an MLP classifier. Five-fold CV was employed to choose one of the five MLPs and one of the hidden layers or the output layer in the CL module and the optimal hyper-parameters of the XGBoost classifier (size of each tree, number of trees, and learning rate) or the MLP classifier (number of hidden layers, number of nodes at each hidden layer, learning rate). The test data were applied to the trained CL model and the classifier, and their class labels were predicted. The predicted results were used to plot an ROC curve and to calculate AUC as the performance measure. The above procedure of randomly splitting data, training and testing the model was repeated 40 times, and the mean and standard deviation of AUCs were calculated, and the Wilcoxon rank-sum test was conducted for performance comparison. Of note, the test data were never used to train the CL module or the classifier.

### CL-based Cox models

Similar to the CL-based classifier, our CLCox model consists of two modules: a CL module for learning feature representations and a Cox module that uses the features from the CL model to predict the prognosis of cancer. We used the Cox proportional hazards model in the Cox module. To predict caner prognosis, we used PFI as the clinical endpoint as recommended by Liu *et. al.* [40]. Let us define the hazard function $h(t)$ of a cancer patient as the instantaneous potential per unit time for the event of cancer recurrence to occur at a time $t$ given that the patient is free of cancer up to $t$. The Cox proportional hazards assumes $h(t)$ in a semi-parametric form [41]: $h(t,{\mathbf x})=h_{0}(t)e^{f_{\theta }({\mathbf x})}$, where we explicitly write $h(t)$ as $h(t,{\mathbf x})$, a function of both $t$ and a feature vector ${\mathbf x}$ which represents gene expression values in our case, $h_{0}(t)$ is a baseline hazard function that does not depend on ${\mathbf x}$, $f_{\theta }({\mathbf x})$ is the output of the model, and $\theta $ represents all the model parameters. We used three existing methods for the Cox model: 1) the regularized Cox regression model with the elastic net (EN) penalty [11], 2) the gradient boosting-based approach implemented with XGBoost [26], and 3) the neural network-based model, Cox-nnet [15]. We refer to the first and the second methods as Cox-EN and Cox-XGB, respectively. The output $f_{\theta }({\mathbf x})$ was modeled with a linear function of ${\mathbf x}$ in Cox-EN, a sum of numerous trees in Cox-XGB, and a neural network in Cox-nnet. The parameters of the Cox model can be estimated by minimizing a loss function equal to the negative log partial likelihood [41, 42], which is given by $L(\theta )=-\sum _{i=1}^{n}{\delta _{i}}\bigl [f_{\theta }({\mathbf x})-\log \bigl ({\sum _{j\in R(t_{i})}\exp (f_{\theta }({\mathbf x}))} \bigr ) \bigr ]$, where $n$ is the number of individuals, $R(t_{i})$ is the set of individuals that are at risk of experiencing an event at time $t_{i}$, and $\delta _{i}$ indicates whether individual $i$ is terminated by an event ($\delta _{i}=1$) or by censoring ($\delta _{i}=0$). Of note, this loss was also used in [13, 15, 18].

To train and test CLCox for each selected type of cancer, we randomly divided the data into a train set with 80% samples and a test set with 20% samples. The CL module was trained with the same procedure as the one described earlier for the CL-based classifier. To train the Cox model, the negative log partial likelihood was used as the loss function. Five-fold CV was used to search over the space of the hyperparameters of the Cox model, five MLPs, and the hidden layer of each MLP whose output was used as the features for training the Cox model. More detailed description of training and testing three Cox models, including implementation of Cox-EN, Cox-XGB, and Cox-nnet and evaluation of Cox models with the c-index and IBS, is described in the Supplemental Text. Of note, the test data were never used in training the CL module or the Cox model.

**Key Points**

- Deep contrastive learning (CL) was applied to learn feature representations from tumor transcriptomes.
- The XGBoost and multilayer perceptron (MLP) classifiers using the features learned by CL significantly outperformed the XGBoost and MLP classifiers using the tumor transcriptome in predicting the recurrence risk of 18 types of cancer.
- The Cox model using the features learned by CL significantly outperformed the Cox model using the tumor transcriptome in predicting the prognosis of 19 types of cancer.
- The prediction of XGBoost classifiers and Cox models trained with TCGA lung and prostate cancer data was validated with the data of two independent cohorts.
- The Cox model using the features learned by CL from the transcriptome significantly outperformed the Cox model using the 16 Oncotype DX genes in predicting the prognosis of breast cancer.

## Supplementary Material

clcox_bb_suppl_v2_bbae544

## Data Availability

All data used in this paper are publicly available. The TCGA pan-cancer RNA-seq and clinical data are available at the GDC website: https://gdc.cancer.gov/about-data/publications/pancanatlas. The CPTAC-3 dataset was obtained from the GDC data portal at https://portal.gdc.cancer.gov/ projects/CPTAC-3. The DKFZ prostate cancer dataset was downloaded from the cBioPortal website at https://www.cbioportal.org/. Five microarray datasets of breast cancer can be accessed from the GEO database at http://www.ncbi.nlm.nih.gov/geo using the GEO accession numbers mentioned in the Datasets section of the Supplemental Text, and another microarray dataset of breast cancer can be accessed from ArrayExpress at http://www.ebi.ac.uk/arrayexpress using the accession number mentioned in the Supplemental Text. The microarray data processed from all six breast cancer datasets by Zhao *et al*. [35] are available at https://miami.app.box.com/s/ylmvqynbtchx5xhof0quaeu9w62mxaca/folder/260013141564. Python codes for training and testing all the models in this paper are publicly available at the following GitHub site: https://github.com/CaixdLab/CL4CaPro. CL-based classifiers and CLCox models trained with the TCGA data are freely accessible at the following Box link: https://miami.box.com/s/ylmvqynbtchx5xhof0quaeu9w62mxaca. We have documented software libraries and provided sample codes for replicating the results in the paper, training and testing new models, and using the trained models to make predictions.
