# Supplementary material for: Deep contrastive learning for predicting cancer prognosis using gene expression values
Source: Brief Bioinform. 2024 Oct 29;25(6):bbae544. doi: 10.1093/bib/bbae544 (PMC11521346; doi:10.1093/bib/bbae544)
Supplement: clcox_bb_suppl_v2_bbae544 [file clcox_bb_suppl_v2_bbae544.pdf]

Supplemental Information

Deep Contrastive Learning for Predicting Cancer Prognosis Using Gene Expression Values

Anchen Sun, Elizabeth J. Franzmann, Zhibin Chen, and Xiaodong Cai

1 Datasets

The RNA-seq and clinical data from The Cancer Genome Atlas (TCGA) were used to train and test models. The datasets are available at the Genomic Data Commons (GDC) of the National Cancer Institute (https://gdc.cancer.gov/about-data/publications/pancanatlas). The RNA-seq dataset contains the expression values of 20,531 genes in 11,069 patients, that have been normalized, batch corrected, platform-corrected by the PanCancer Atlas consortium [1]. Similarly, the corresponding clinical data of the cancer patients have been curated by the PanCancer Atlas consortium [2]. Non-cancer samples were removed, and genes with missing or negative expression values were excluded. Patient samples with missing PFI were also removed. Let the expression value of a gene be denoted as x, then, it was log-transformed as $\log_2(1+x)$. The PFI and the censoring information were extracted from the clinical data. Of note, we used PFI instead of the overall survival (OS) time in our analysis, because PFI is generally a better clinical endpoint choice than OS [2]. We select 18 types of cancer that have > 250 samples with both RNA-seq and PFI data, and glioblastoma multiforme (GBM) that has only 160 patients but a relatively large number of uncensored samples (127), to build and test models for predicting their prognosis. The 18 types of cancer are bladder urothelial carcinoma (BLCA), breast invasive carcinoma (BRCA), cervical squamous cell carcinoma and endocervical adenocarcinoma (CESC), colon adenocarcinoma (COAD), head and neck squamous cell carcinoma (HNSC), kidney renal clear cell carcinoma (KIRC), kidney renal papillary cell carcinoma (KIRP), brain lower grade glioma (LGG), liver hepatocellular carcinoma (LIHC), lung adenocarcinoma (LUAD), lung squamous cell carcinoma (LUSC), ovarian serous cystadenocarcinoma (OV), prostate adenocarcinoma (PRAD), sarcoma (SARC), skin cutaneous melanoma (SKCM), stomach adenocarcinoma (STAD), thyroid carcinoma (THCA), and uterine corpus endometrial carcinoma (UCEC).

The data of CPTAC-3 [3] and a prostate cancer data set named DKFZ [4] were used to validate the models trained with the TCGA data. The CPTAC-3 dataset was downloaded from the GDC data portal. It contains the RNA-seq and clinical data of five types of cancer: LUAD, LUSC, renal cell carcinoma, glioblastoma, and UCEC. Only LUAD and LUSC are among the 18 types of cancer for which a classifier was trained with the TCGA data to predict recurrence risk as described late. Therefore, we extracted RNA-seq data and the PFI information of these two types of cancer, which yielded 206 and 102 samples for LUAD and LUSC, respectively. The DKFZ dataset was downloaded from cBioPortal. It contained RNA-seq and PFI of 105 prostate cancer patients.

A collection of 6 breast cancer microarray datasets was used to train and test Cox models that use the expression values of 16 genes of Oncotype DX [5] or all genes in the data set as the input. This collection of data was compiled by van Vliet *et. al.* [6] and was used by Zhao *et. al.* [7] to assess the performance of 9 gene signatures for predicting the prognosis of breast cancer. These 6 datasets were obtained with Affymetrix Human Genome HG-U133A arrays. Five of them can be accessed from the Gene Expression Omnibus (GEO) with the following accession numbers: GSE6532 [8], GSE3494 [9], GSE1456 [10], GSE7390 [11], and GSE2603 [12]; the other one can be accessed from ArrayExpress with the accession number E-TABM-158 [13]. We obtained from the author of [7] the preprocessed and normalized gene expression data and clinical data that include DMFS time. The pooled dataset contains a total of 947 patients, and we extracted 687 ER+ samples. It has the expression values of 20,3507 probes; we mapped these probes to 13,235 genes and calculated the expression value of each gene as the mean of expression values of all probes mapped to the gene.

2 Implementation of Cox-EN, Cox-XGB, and Cox-nnet

Cox-EN was implemented with Python module scikit-survival [14], and Cox-XGB was implemented with the software package XGBoost [15]. The regularization term of Cox-EN is $\lambda(\alpha\|\boldsymbol{\theta}\|_1 + (1-\alpha)/2\|\boldsymbol{\theta}\|_2^2)$, where $\|\boldsymbol{\theta}\|_1$ and $\|\boldsymbol{\theta}\|_2$ are the ℓ_1- and ℓ_2-norm of $\boldsymbol{\theta}$, respectively, and $\lambda > 0$ and $0 \leq \alpha \leq 1$ are two hyperparameters. Five-fold cross validation was employed to search over the two pre-specified sets of values for λ and α to determine their optimal values. The XGBoost model has a number of hyperparameters. We used the default values of most hyperparameters except for the maximum tree depth, parameter λ for ℓ_2-regularization, and subsample, which were determined with five-fold CV.

We downloaded the Python code of Cox-nnet [16] and updated several Python functions so that the code could run under Python 3.11 and PyTorch 2.0. We trained the ANNs of Cox-nnet, that have one or two fully connected (FC) hidden layers with sigmoid action function, with ℓ_2-regularization by minimizing the Cox loss function, which is the negative log partial likelihood. The hyperparameters include the number of hidden layers (1, 2), the number of nodes at hidden layer(s), the weight of ℓ_2-regularization, and the learning rate. Five-fold CV was employed to search over the set of hyper-parameter values to determine the optimal neural network.

3 Evaluation of Cox models

The performance of Cox models on prognosis prediction was evaluated with three commonly used metrics: the Harrell's concordance index (C-index) [17], the integrated Brier score (IBS) [18], and the time-dependent ROC curves [19, 20, 21]. The C-index is defined as the ratio of the concordant pairs of predictions and all comparable pairs of patients, where a pair of patients is called concordant if the risk of the event predicted by a model is lower for the patient who experiences the event at a later time point. A C-index value of 0.5 indicates random prediction. The value of C-index increases when the prediction accuracy increases, and a value of 1.0 indicates perfect prediction,

where all pairs are concordant.

Suppose that the test set contains m samples and let t_i and δ_i, $i = 1, 2, \cdots, m$ be the PFI and the indicator of the censoring status, respectively, of the ith sample. The Brier score at time t under random censorship can be estimated as [18, 22]

$$BS(t) = \frac{1}{m} \sum_{i=1}^{m} \left\{ \frac{(0 - \hat{S}_i(t))^2 1_{t_i \leq t, \delta_i = 1}}{\hat{G}(t_i)} + \frac{(1 - \hat{S}_i(t))^2 1_{t_i > t}}{\hat{G}(t)} \right\},$$

where $S_i(t)$ is the estimated survival function of individual i, and $\hat{G}(t)$ is the Kaplan-Meier estimated of the censoring distribution. The survival function $S_i(t)$ can be estimated as $\hat{S}_i(t) = e^{-\hat{H}_0(t) \exp(f_\theta(\mathbf{x}_i))}$, where $\hat{H}_0(t)$ is the cumulative baseline hazard function estimated from the training data using the Breslow estimator [23]:

$$\hat{H}_0(t) = \sum_{j: t_j \leq t} \left\{ \frac{\delta_j}{\sum_{k \in \mathcal{R}(t_j)} \exp(f_\theta(\mathbf{x}_k))} \right\}.$$

The IBS is then calculated as IBS $= \frac{1}{T} \int_0^T BS(t) dt$, where T is determined as follows. It is seen from the formula of $\hat{H}_0(t)$ that we can get $\hat{H}_0(t)$ up to the maximum uncensored t_j in the training data. However, the size of the set $\mathcal{R}(t_j)$ decreases when t_j increases. For those t where the formula of $\hat{H}_0(t)$ contains $\mathcal{R}(t_j)$ whose size is small, $\hat{H}_0(t)$ may not be accurate. To avoid this problem, we define $t_{\max}$ as the maximum uncensored t_j in the training data where the size of $\mathcal{R}(t_j)$ is greater than or equal to 20. Also, we define $\tilde{t}_{max}$ as the maximum t_j in the test data. Then, we set $T = \min\{t_{\max}, \tilde{t}_{\max}\}$.

The time-dependent ROC curve at a specific time t is determined by calculating cumulative sensitivity and dynamic specificity at t [19, 20, 21] . Specifically, at a time point t, each individual is classified as a case or control. A case is defined as any individual experiencing the event between $t = 0$ and time t, and a control as an individual remaining event-free at time t. The cumulative sensitivity is the probability that an individual has a survival probability $S(t)$ less than a threshold c among the individuals who experienced the event before time t, and the dynamic specificity is the probability that an individual has a $S(t)$ greater than or equal to c among those event-free individuals beyond time t. The estimate of $S(t)$ for the ith individual, $\hat{S}_i(t)$, is also used in computing the IBS, as described earlier. We used the IPCW estimator [19, 20] for calculating the sensitivity and the specificity, and the ROC curve was then obtained by plotting sensitivity versus 1-specificity.

As mentioned earlier, we randomly split data into a training set and a test set. We used the training set to train the model, used the trained model to make prediction on the test set, and then computed the two performance metrics. We repeated this process 40 times, each time with a random seed for splitting the data. We used results of 40 repeats to produce box plots of the three performance metrics, to compute their mean and standard deviation, and to perform Wilcoxon rank-sum test for performance comparison.

4 Pooling data of different types of cancer for model training

We identified the following 8 groups of cancers from clustering analysis of TCGA RNA-seq data by Hoadley *et al.* [1]: a group with squamous morphology (BLCA, CESC, ESCA, HNSC, and LUSC), glioma tumors (GBM and LGG), melanomas of the skin and eye (SKCM and UVM), clear cell and papillary renal carcinomas (KIRC and KIRP), hepatocellular and cholangiocarcinomas (LIHC and CHOL), a gastrointestinal group (COAD, READ, non-squamous ESCA, READ, and STAD), a digestive system group (PAAD, STAD, and a few ESCA), and two mixed lung cancer groups (LUAD and LUSC). For each group of cancers, we split the data into a training set with 80% samples and a test set with 20% samples, and used the training data of all types of cancers in the group to train the MLP in the CL module. Then, for each type of cancer in the group, we used the features output from the MLP with the samples in the training set of that type of cancer to train a XGBoost classifier or a Cox model. We again considered three Cox models: Cox-EN, Cox-XGB, and Cox-nnet. Of note, the test data were not used in training the CL model, the classifier, or the Cox model.

5 Model validation with independent cohorts

We used CPTAC-3 lung cancer (LUAD and LUSC) data [3] and DKFZ prostate cancer data [4] to validate the Cox models and classifiers trained with TCGA LUAD, LUSC, and PRAD data. As mentioned earlier, the CPTAC-3 dataset contains 206 LUAD patients and 102 LUSC patients, and the DKFZ dataset contains 105 PRAD patients, which are used to validate Cox models trained with TCGA data. To validate the classifiers, we divided data samples for each type of cancer into a high-risk group (PFI $<$ 3 years) and a low-risk group (PFI $>$ 3 years). This resulted in 47, 125, 23 samples in the high-risk group, and 45, 53, 47 samples in the low-risk group for CPTAC-3 LUSC, CPTAC-3 LUAD, and DKFZ PRAD datasets, respectively. Of note, the samples with a censored PFI $<$ 3 years had to be discarded, because their class labels were unknown.

Both CPTAC-3 and DKFZ RNA-seq data contain reads per kilobase million (RPKM) values for each gene. But the pan-cancer TCGA RNA-seq data that we used contain the read counts for each gene (per private communication with the first author of [1]). Therefore, we converted RPKM values to read counts per gene by multiplying the RPKM value of a gene by the length of the gene.

We used expression values of housekeeping genes to normalize gene expression values in different datasets. Based on RNA-seq data, Eisenberg and Levanon identified eleven genes that were highly uniform and strongly expressed in all human tissues [24]. Ten out of the eleven genes are present in the TCGA dataset, and they are C1orf43, CHMP2A, GPI, PSMB2, PSMB4, RAB7A, REEP5, SNRPD3, VCP, and VPS29. We computed the average expression values of these 10 genes in the TCGA and CPTAC-3 LUAD datasets, which are denoted as E_t and E_c, respectively. Then, the expression values of all genes in the CPTAC-3 LUAD dataset were multiplied by a normalization factor E_t/E_c. Similarly, we used the 10 housekeeping genes to normalize the gene expression values of the LUSC datasets. We found that the variances of the expression values of the 10 housekeeping genes in the TCGA PRAD dataset had relatively large variations. Therefore, we selected the three genes, VCP, RAB7A, and GPI, that had smallest variance in their expression values, and used

the average expression values of these three genes in the TCGA PRAD and DKFZ datasets to normalize gene expression values.

Not all genes in the TCGA dataset are present in the CPTAC-3 and DKFZ datasets. Specifically, 534, 561, and 1,215 genes in TCGA LUAD, LUSC, and PRAD data were missing in CPTAC-3 LUAD, CPTAC-3 LUSC, and DKFZ data, respectively. Of note, the number of genes in TCGA LUAD and LUSC data is 20,531, but the number of genes in TCGA PRAD data is 16,136, because some genes with missing expression values were removed. Therefore, we extracted expression values of 19,997, 19,970, and 14,921 genes in CPTAC-2 LUAD and LUSC, and DKFZ datasets, respectively, that are present in the corresponding TCGA datasets. The expression values of these genes were $\log_2$-transformed as done to the TCGA gene expression values, and expression values of the missing genes were set to zero. Gene expression values of the CPTAC-3 and DKFZ data were input to the Cox models and classifiers trained with the corresponding TCGA LUAD, LUSC, and PRAD data, and the outputs of each model were used to compute performance metrics including c-index for Cox models and AUC for classifiers.

6 Comparison of Cox models with Oncotype DX genes and all available genes

We used gene expression and DMFS data of 687 ER+ breast patients in a collection of 6 breast cancer datasets [6, 7] described earlier to train Cox models. Expression values of each gene were normalized by subtracting the average value of the following five housekeeping genes: ACTB, GAPDH, RPLPO, GUS, and TFRC [5]. The dataset was randomly split into a training set with 80% samples and a test set with 20% samples. We trained Cox-EN, Cox-XGB, Cox-nnet, CLCox-EN, CLCox-XGB, and CLCox-nnet models with the 16 genes of Oncotype DX, and also trained these six models with all 13,235 genes in the data set, and then determined the c-index of each model using the test data. This process of random splitting of data, training, and testing was repeated 40 times.

Oncotype DX uses the RS to divide patients into three groups: a high-risk group (RS$\geq$ 31), a medium-risk group (18$\leq$ RS$<$ 31), and a low-risk group (RS$<$ 18) [5]. Clinical trials showed that patients in different groups had different DMFS probabilities [5, 25]. We used the HR predicted by a Cox model to divide patients into three groups. Specifically, we ranked HRs of all patients, predicted by Cox-EN with the training data, in the descent order, and followed the approach in [7, 5] to determine two cutoff values c_1 (= 73th percentile of HRs) and c_2 (= 51st percentile of HRs). We divided the patients in the test data into three groups: a high-risk group (HR$\geq c_1$), a medium-risk group ($c_2 \leq$ HR$< c_1$), and a low-risk group (HR$< c_1$), and then compared the KM curves of the patients in the three groups.

References

[1] Hoadley KA, Yau C, Hinoue T, Wolf DM, Lazar AJ, Drill E, et al. Cell-of-origin patterns dominate the molecular classification of 10,000 tumors from 33 types of cancer. Cell.

2018;173(2):291-304.

[2] Liu J, Lichtenberg T, Hoadley KA, Poisson LM, Lazar AJ, Cherniack AD, et al. An integrated TCGA pan-cancer clinical data resource to drive high-quality survival outcome analytics. Cell. 2018;173(2):400-16.

[3] Edwards NJ, Oberti M, Thangudu RR, Cai S, McGarvey PB, Jacob S, et al. The CPTAC data portal: a resource for cancer proteomics research. Journal of Proteome Research. 2015;14(6):2707-13.

[4] Gerhauser C, Favero F, Risch T, Simon R, Feuerbach L, Assenov Y, et al. Molecular evolution of early-onset prostate cancer identifies molecular risk markers and clinical trajectories. Cancer cell. 2018;34(6):996-1011.

[5] Paik S, Shak S, Tang G, Kim C, Baker J, Cronin M, et al. A multigene assay to predict recurrence of tamoxifen-treated, node-negative breast cancer. New England Journal of Medicine. 2004;351(27):2817-26.

[6] van Vliet MH, Reyal F, Horlings HM, van de Vijver MJ, Reinders MJ, Wessels LF. Pooling breast cancer datasets has a synergetic effect on classification performance and improves signature stability. BMC Genomics. 2008;9(375):1-22.

[7] Zhao X, Rødland EA, Sørlie T, Vollan HKM, Russnes HG, Kristensen VN, et al. Systematic assessment of prognostic gene signatures for breast cancer shows distinct influence of time and ER status. BMC Cancer. 2014;14(211):1-12.

[8] Loi S, Haibe-Kains B, Desmedt C, Lallemand F, Tutt AM, Gillet C, et al. Definition of clinically distinct molecular subtypes in estrogen receptor-positive breast carcinomas through genomic grade. Journal of Clinical Oncology. 2007;25(10):1239-46.

[9] Miller LD, Smeds J, George J, Vega VB, Vergara L, Ploner A, et al. An expression signature for p53 status in human breast cancer predicts mutation status, transcriptional effects, and patient survival. Proceedings of the National Academy of Sciences. 2005;102(38):13550-5.

[10] Pawitan Y, Bjöhle J, Amler L, Borg AL, Egyhazi S, Hall P, et al. Gene expression profiling spares early breast cancer patients from adjuvant therapy: derived and validated in two population-based cohorts. Breast Cancer Research. 2005;7(6):1-12.

[11] Desmedt C, Piette F, Loi S, Wang Y, Lallemand F, Haibe-Kains B, et al. Strong time dependence of the 76-gene prognostic signature for node-negative breast cancer patients in the TRANSBIG multicenter independent validation series. Clinical Cancer Research. 2007;13(11):3207-14.

[12] Minn AJ, Gupta GP, Siegel PM, Bos PD, Shu W, Giri DD, et al. Genes that mediate breast cancer metastasis to lung. Nature. 2005;436(7050):518-24.

[13] Chin K, DeVries S, Fridlyand J, Spellman PT, Roydasgupta R, Kuo WL, et al. Genomic and transcriptional aberrations linked to breast cancer pathophysiologies. Cancer Cell. 2006;10(6):529-41.

[14] Pölsterl S. scikit-survival: A Library for Time-to-Event Analysis Built on Top of scikit-learn. Journal of Machine Learning Research. 2020;21(212):1-6.

[15] Chen T, Guestrin C. XGBoost: A scalable tree boosting system. In: Proceedings of the 22nd ACM SIGKDD International Conference on Knowledge Discovery and Data Mining; 2016. p. 785-94.

[16] Ching T, Zhu X, Garmire LX. Cox-nnet: an artificial neural network method for prognosis prediction of high-throughput omics data. PLoS Computational Biology. 2018;14(4):e1006076.

[17] Harrell Jr FE, Lee KL, Mark DB. Multivariable prognostic models: issues in developing models, evaluating assumptions and adequacy, and measuring and reducing errors. Statistics in Medicine. 1996;15(4):361-87.

[18] Graf E, Schmoor C, Sauerbrei W, Schumacher M. Assessment and comparison of prognostic classification schemes for survival data. Statistics in Medicine. 1999;18(17-18):2529-45.

[19] Uno H, Cai T, Tian L, Wei LJ. Evaluating prediction rules for t-year survivors with censored regression models. Journal of the American Statistical Association. 2007;102(478):527-37.

[20] Blanche P, Dartigues JF, Jacqmin-Gadda H. Estimating and comparing time-dependent areas under receiver operating characteristic curves for censored event times with competing risks. Statistics in medicine. 2013;32(30):5381-97.

[21] Kamarudin AN, Cox T, Kolamunnage-Dona R. Time-dependent ROC curve analysis in medical research: current methods and applications. BMC medical research methodology. 2017;17:1-19.

[22] Fotso S, et al.. PySurvival: Open source package for Survival Analysis modeling; 2019. Available from: https://www.pysurvival.io/.

[23] Breslow NE. Analysis of survival data under the proportional hazards model. International Statistical Review/Revue Internationale de Statistique. 1975;43(1):45-57.

[24] Eisenberg E, Levanon EY. Human housekeeping genes, revisited. TRENDS in Genetics. 2013;29(10):569-74.

[25] Paik S, Tang G, Shak S, Kim C, Baker J, Kim W, et al. Gene expression and benefit of chemotherapy in women with node-negative, estrogen receptor–positive breast cancer. Journal of Clinical Oncology. 2006;24(23):3726-34.

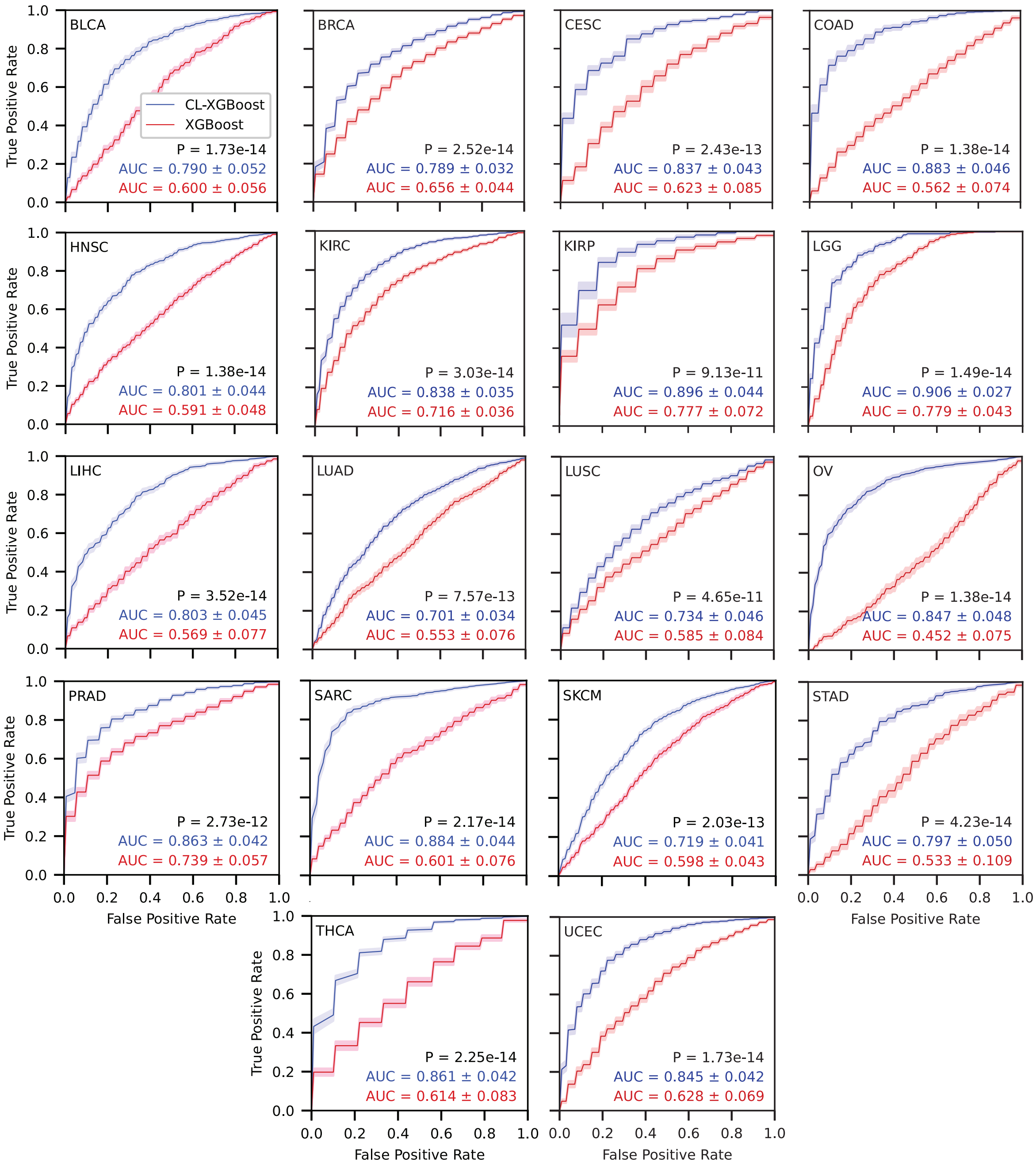

Figure S1: **ROC curves and AUC of XGBoost classifiers with and without contrastive learning for classifying cancer patients into a high or low risk group of disease recurrence.** A cut-off value of 3 years of PFI was used to define two risk groups.

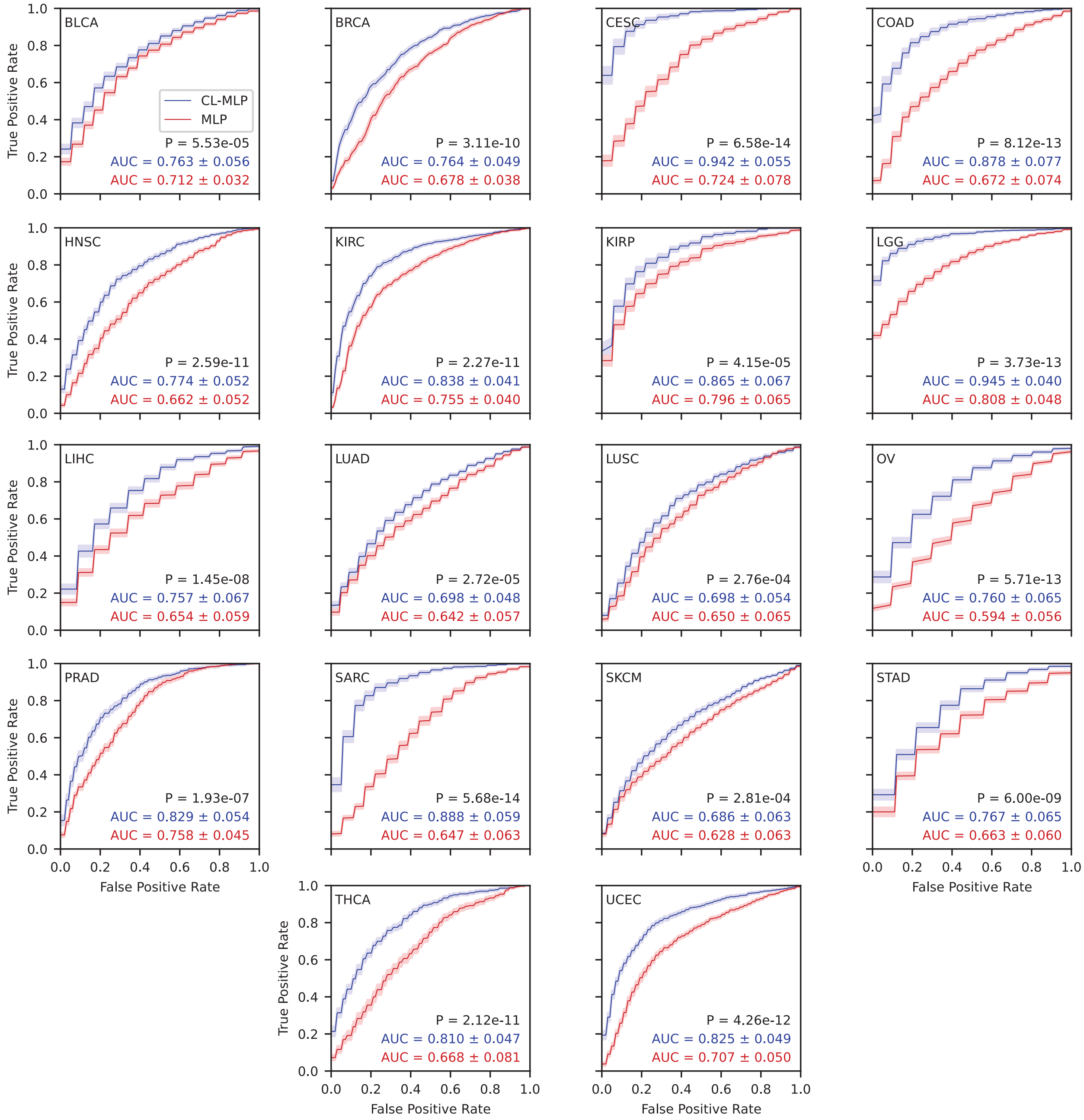

Figure S2: **ROC curves and AUC of MLP classifiers with and without contrastive learning for classifying cancer patients into a high or low risk group of disease recurrence.** A cut-off value of 3 years of PFI was used to define two risk groups.

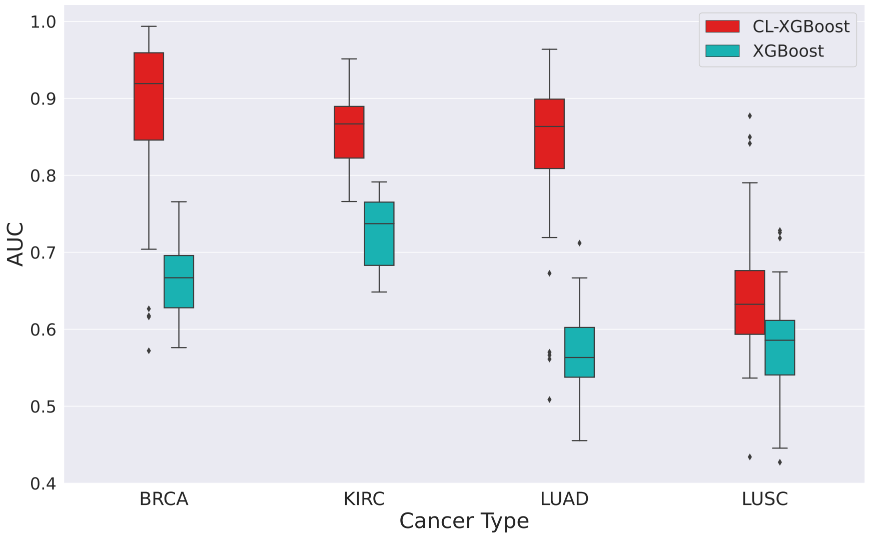

Figure S3: **Performance of XGBoost and CL-XGBoost classifier for four types of cancer.** A cut-off value of 5 (2) years of PFI was used to define two risk groups for BRCA and KIRC (LUAD and LUSC).

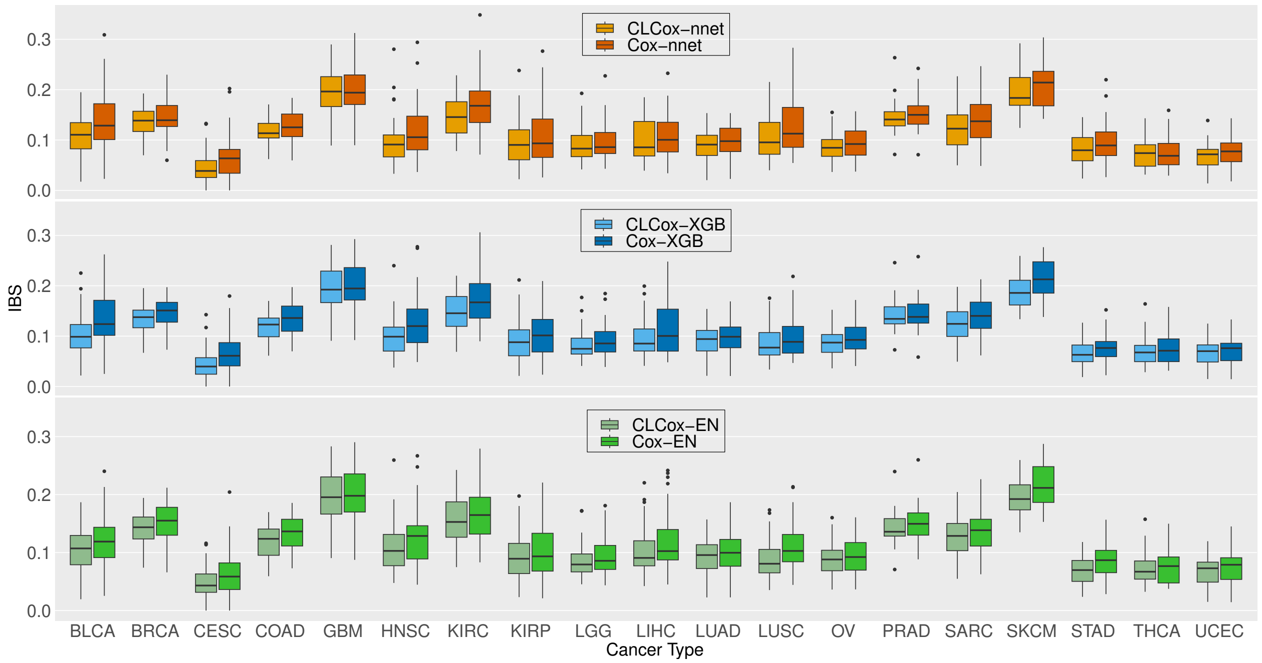

Figure S4: **IBSs of Cox models, each of which was trained with the data of one of 19 types of cancer.**

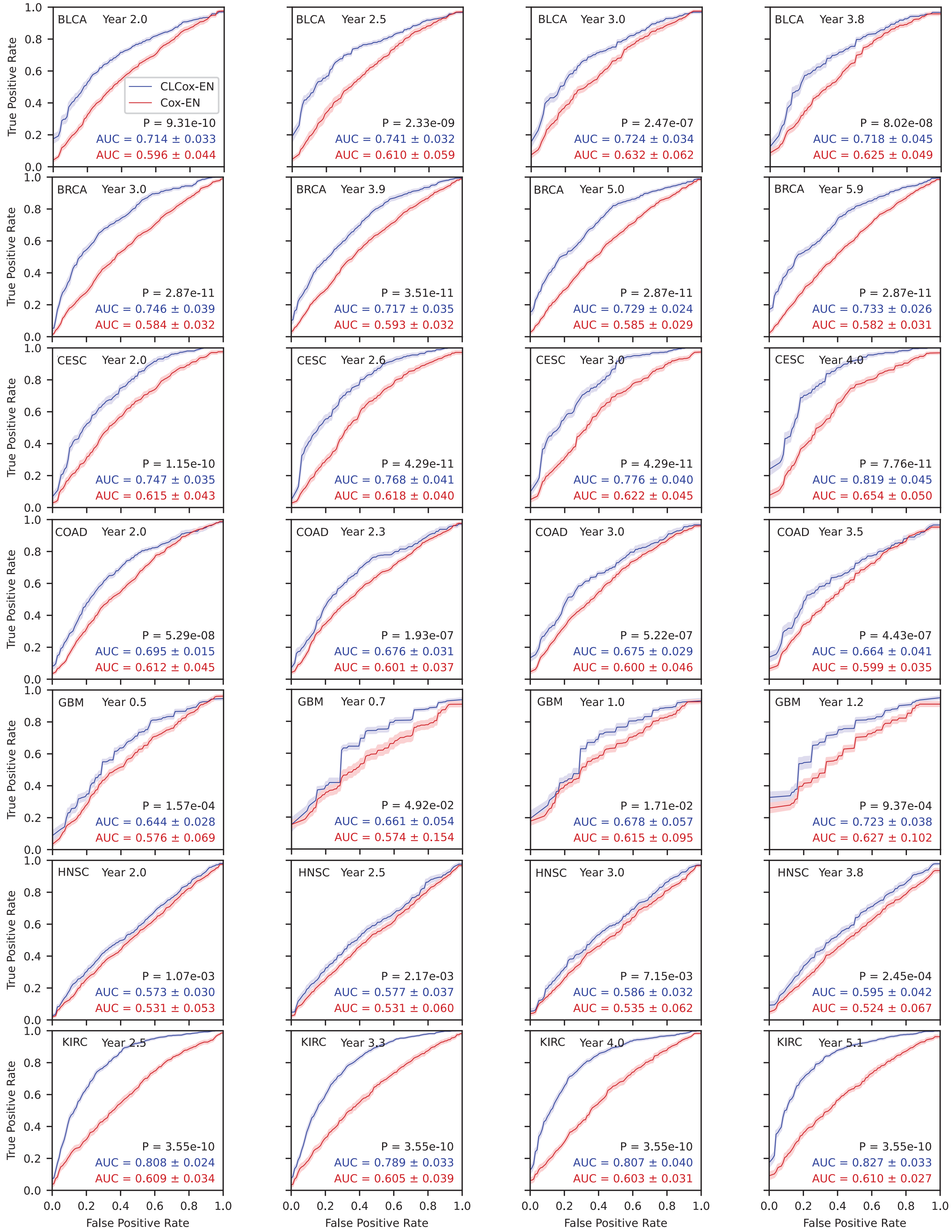

Figure S5: **Time-dependent ROC curves of Cox-EN with and without contrastive learning for the following 7 types of cancer: BLCA, BRCA, CESC, COAD, GBM, HNSC, and KIRC.**

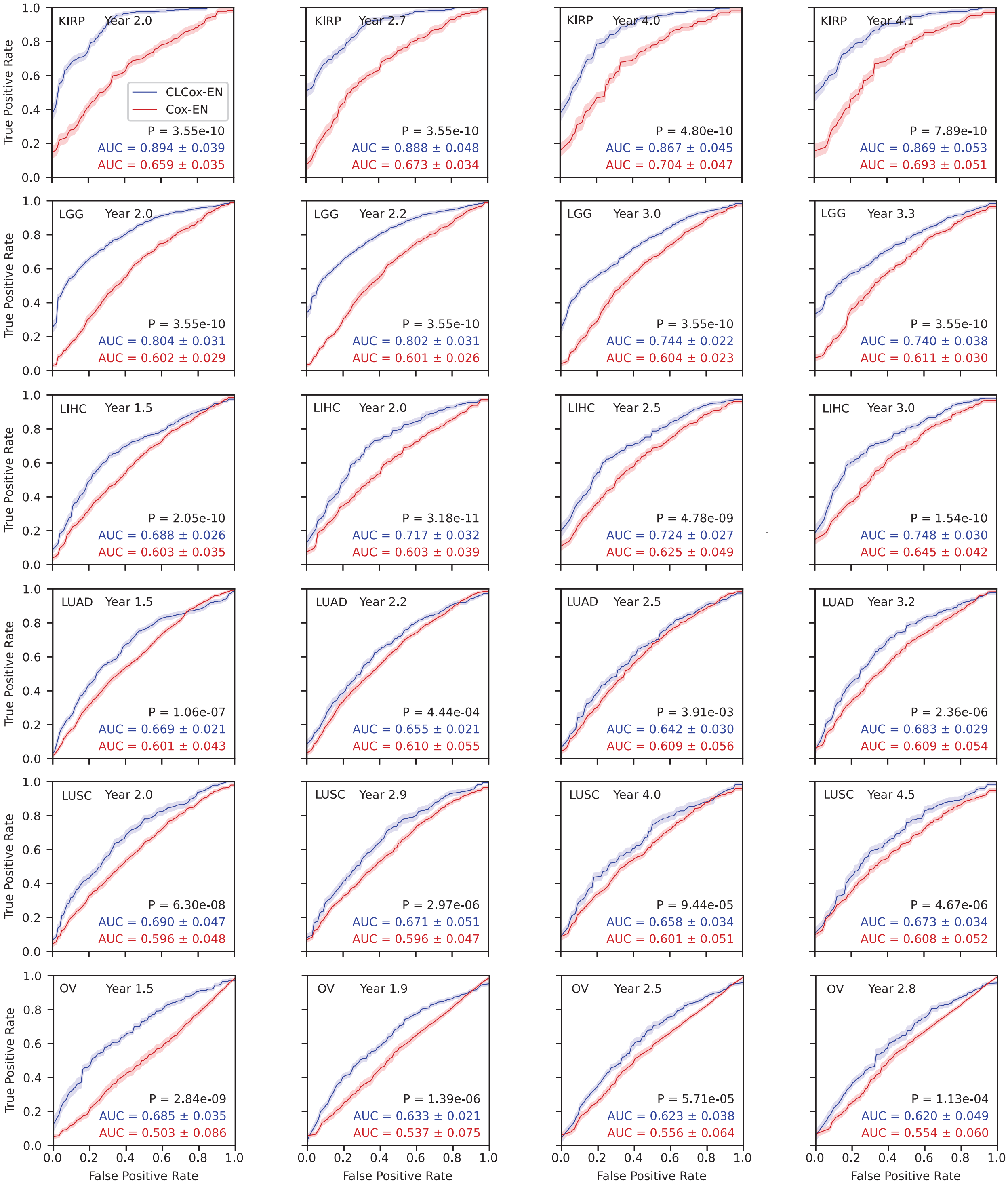

Figure S6: **Time-dependent ROC curves of Cox-EN with and without contrastive learning for the following 6 types of cancer: KIRP, LGG, LIHC,LUAD, LUSC, and OV,**

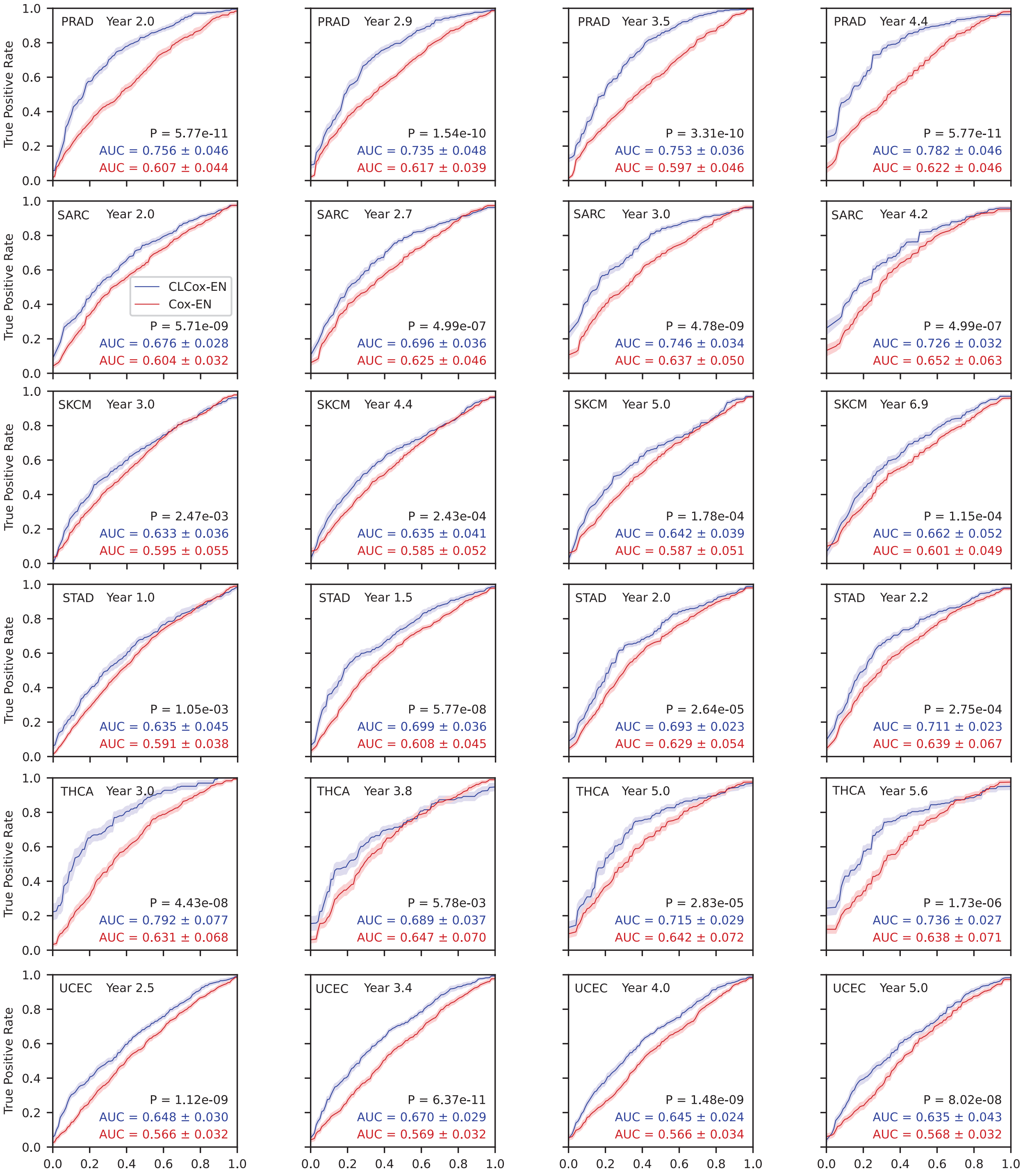

Figure S7: **Time-dependent ROC curves of Cox-EN with and without contrastive learning for the following 6 types of cancer: PRAD, SARC, SKCM, STAD, THCA, UCEC**

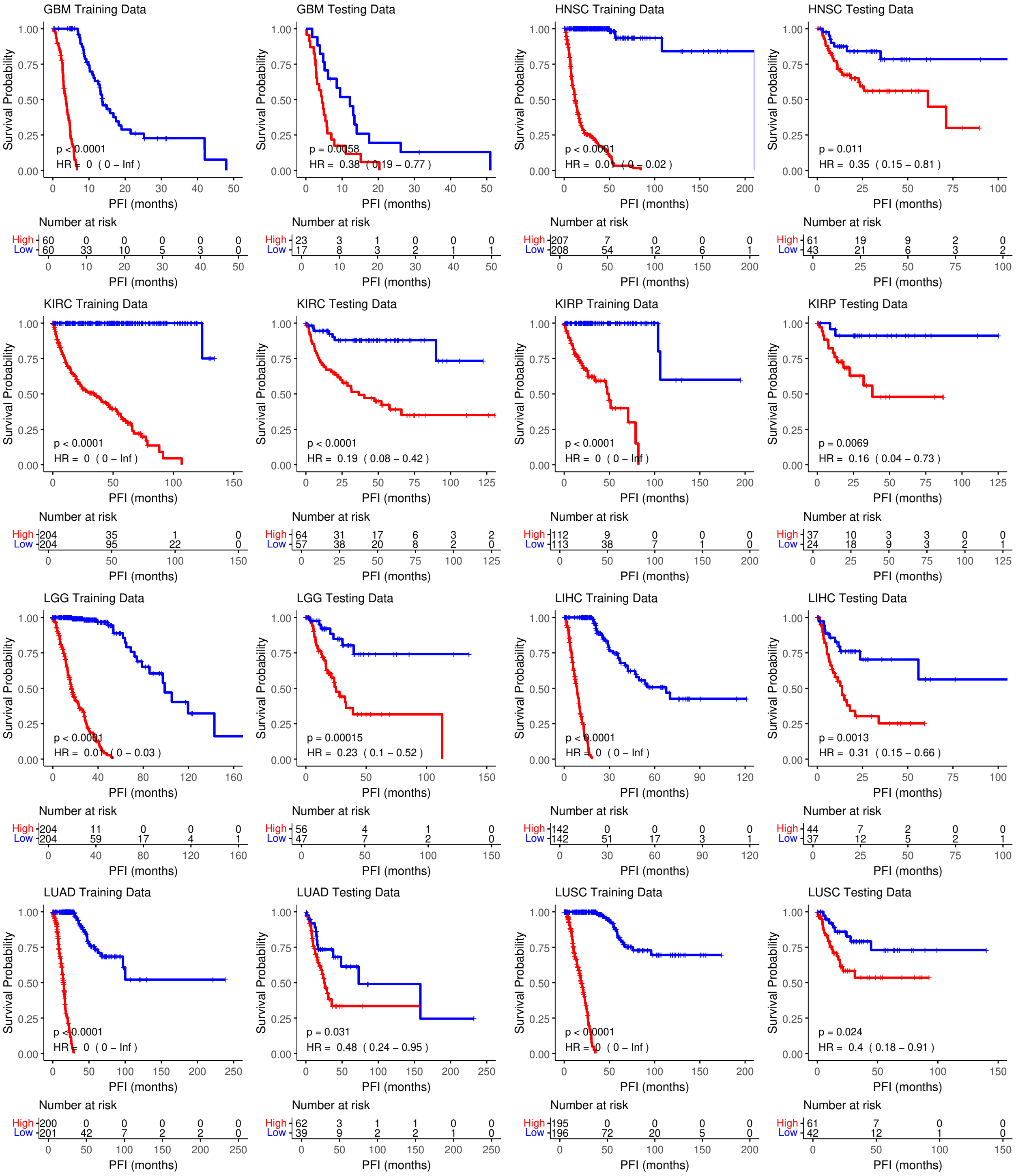

Figure S8: **KM-curves for the two groups of patients stratified by the median HR predicted by the CLCox-XGB model using the training data.** The two groups are a poorly prognostic group (red) with patients' HR greater than the median HR and a better prognostic group (blue) with patients' HR less than the median HR.

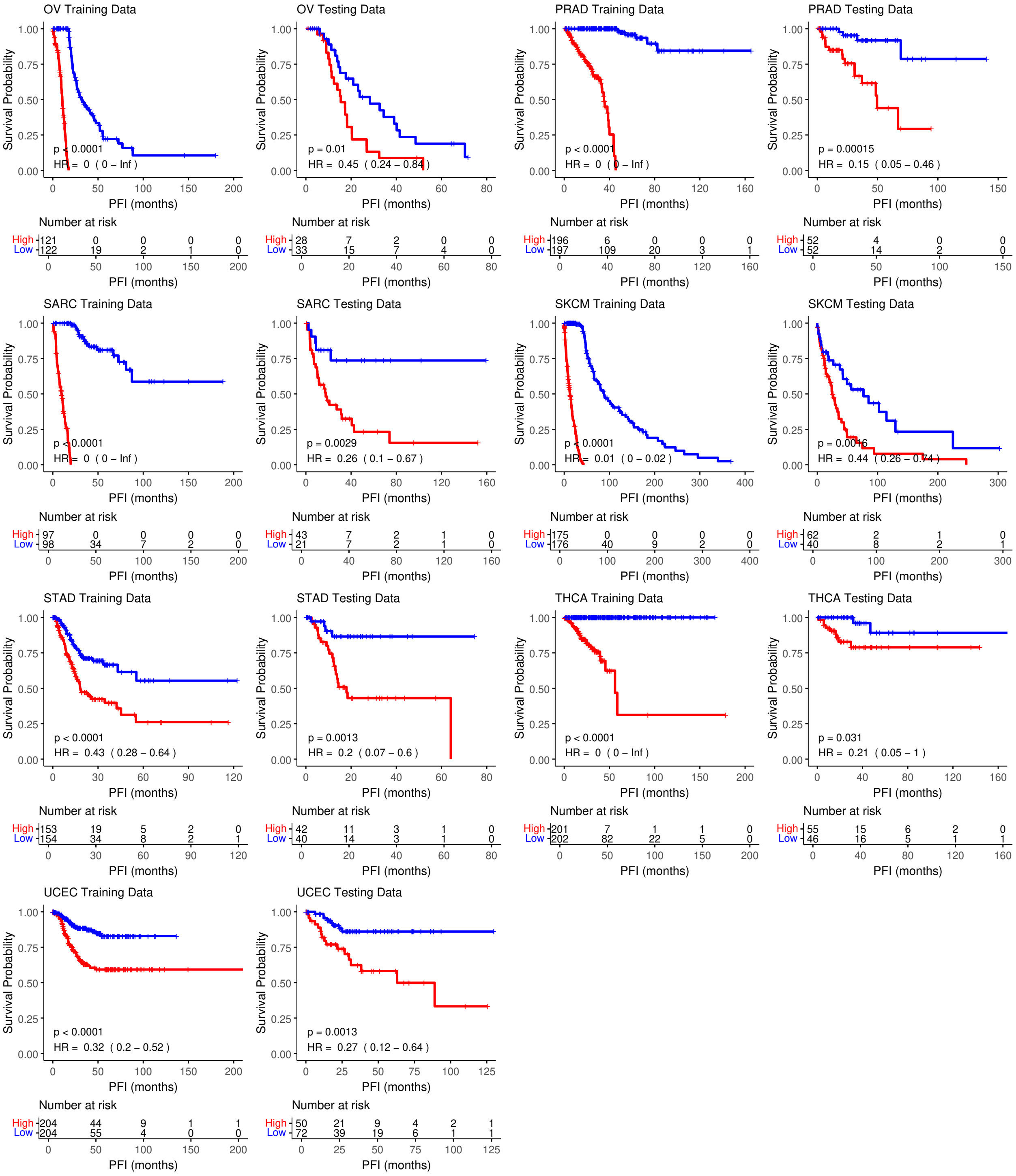

Figure S9: **KM-curves for the two groups of patients stratified by the median HR predicted by the CLCox-XGB model using the training data.** The two groups are a poorly prognostic group (red) with patients' HR greater than the median HR and a better prognostic group (blue) with patients' HR less than the median HR.

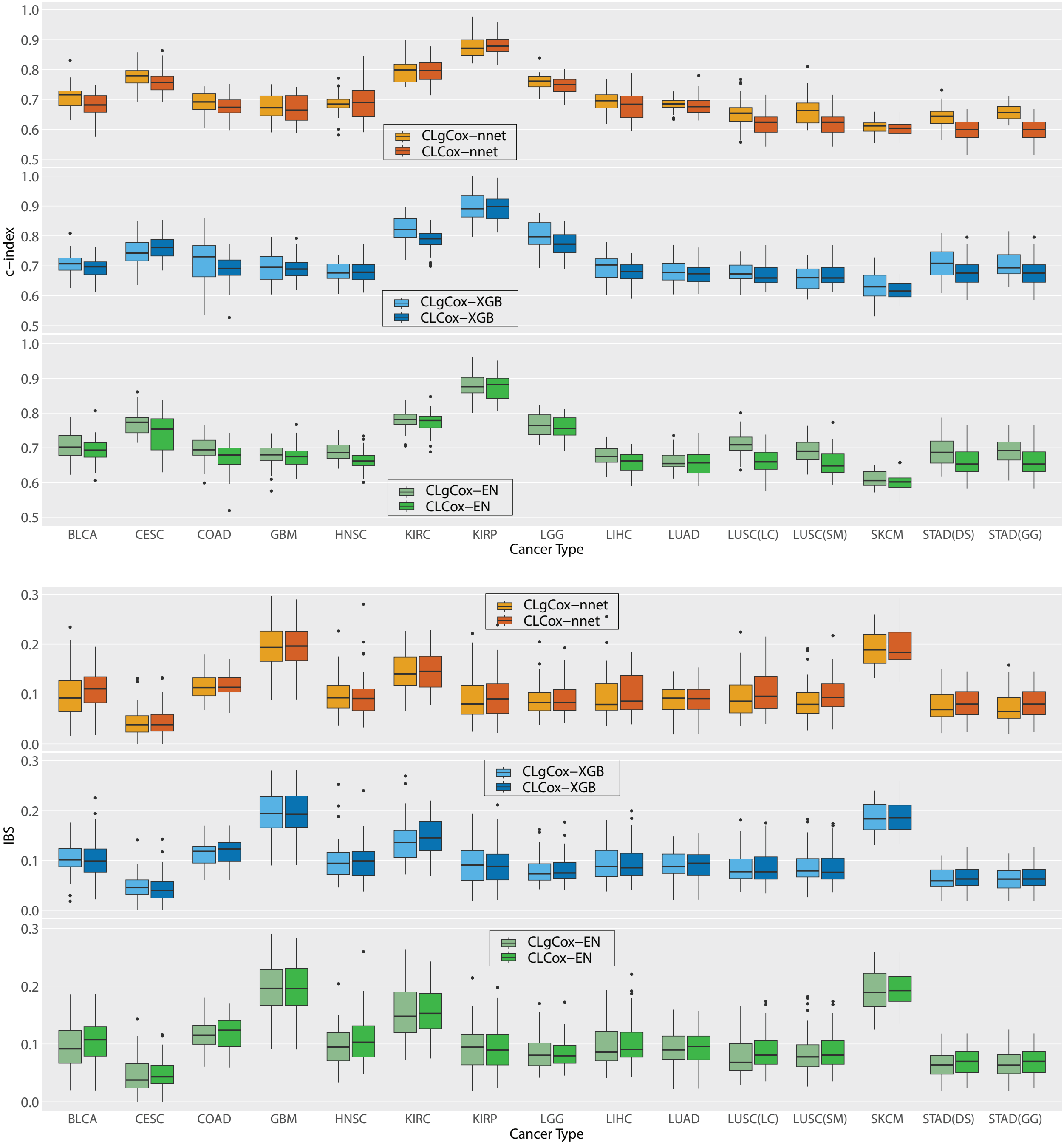

Figure S10: **Performance of Cox models trained with pooled data of a group of cancer types and data of a single type of cancer.** CLgCox-EN, CLgCox-XGB, and CLgCox-nnet stands for the methods trained with pooled data. LUSC(SM) and LUSC(LC) represent LUSC trained within the group with squamous morphology and the mixed lung cancer group, respectively. STAD(GG) and STAD(DS) represent STAD trained within the gastrointestinal group and the digestive system group, respectively.

Table S1: **C-indexes of six Cox models in predicting the hazard ratio of 19 types of cancer.**

Cancer Type	CLCox-nnet	Cox-nnet	p-value	CLCox-XGB	Cox-XGB	p-value	CLCox-EN	Cox-EN	p-value
BLCA	0.681 ± 0.040	0.603 ± 0.067	2.4e-07	0.693 ± 0.037	0.610 ± 0.051	2.7e-10	0.692 ± 0.035	0.653 ± 0.041	8.3e-05
BRCA	0.772 ± 0.044	0.720 ± 0.044	5.6e-06	0.753 ± 0.047	0.708 ± 0.053	2.2e-04	0.737 ± 0.040	0.672 ± 0.058	7.6e-07
CESC	0.756 ± 0.037	0.679 ± 0.050	9.4e-10	0.761 ± 0.040	0.664 ± 0.062	1.1e-10	0.742 ± 0.058	0.679 ± 0.055	2.3e-05
COAD	0.677 ± 0.033	0.619 ± 0.042	1.4e-08	0.688 ± 0.048	0.613 ± 0.052	1.7e-08	0.671 ± 0.041	0.605 ± 0.044	4.1e-09
GBM	0.670 ± 0.048	0.600 ± 0.041	7.8e-09	0.690 ± 0.037	0.616 ± 0.028	4.9e-12	0.677 ± 0.035	0.607 ± 0.046	3.1e-08
HNSC	0.696 ± 0.058	0.643 ± 0.043	1.4e-04	0.682 ± 0.038	0.607 ± 0.043	5.4e-10	0.663 ± 0.028	0.602 ± 0.047	2.0e-08
KIRC	0.795 ± 0.038	0.751 ± 0.049	3.4e-05	0.787 ± 0.040	0.734 ± 0.047	2.3e-06	0.772 ± 0.031	0.742 ± 0.033	8.0e-05
KIRP	0.879 ± 0.033	0.811 ± 0.049	2.3e-09	0.894 ± 0.048	0.817 ± 0.064	5.6e-07	0.880 ± 0.039	0.827 ± 0.054	1.9e-05
LGG	0.746 ± 0.027	0.708 ± 0.027	1.3e-07	0.773 ± 0.039	0.733 ± 0.035	4.0e-05	0.758 ± 0.032	0.717 ± 0.038	1.0e-05
LIHC	0.680 ± 0.049	0.655 ± 0.070	1.3e-01	0.678 ± 0.034	0.625 ± 0.051	3.1e-06	0.658 ± 0.031	0.619 ± 0.038	8.0e-06
LUAD	0.678 ± 0.030	0.617 ± 0.039	1.8e-10	0.672 ± 0.036	0.595 ± 0.044	2.7e-10	0.657 ± 0.035	0.598 ± 0.037	2.6e-09
LUSC	0.619 ± 0.040	0.549 ± 0.035	1.4e-10	0.670 ± 0.040	0.633 ± 0.046	1.0e-03	0.661 ± 0.040	0.593 ± 0.046	1.4e-08
OV	0.664 ± 0.051	0.584 ± 0.052	9.8e-09	0.643 ± 0.035	0.550 ± 0.030	6.6e-14	0.627 ± 0.032	0.565 ± 0.038	2.1e-10
PRAD	0.735 ± 0.041	0.703 ± 0.041	9.3e-04	0.780 ± 0.043	0.732 ± 0.057	3.4e-04	0.762 ± 0.039	0.695 ± 0.051	2.0e-08
SARC	0.698 ± 0.034	0.634 ± 0.041	5.5e-09	0.691 ± 0.039	0.623 ± 0.044	3.9e-09	0.678 ± 0.038	0.638 ± 0.043	1.8e-04
SKCM	0.601 ± 0.024	0.567 ± 0.037	2.2e-05	0.617 ± 0.028	0.559 ± 0.039	1.9e-09	0.602 ± 0.027	0.560 ± 0.026	7.8e-09
STAD	0.599 ± 0.034	0.536 ± 0.044	1.4e-08	0.676 ± 0.047	0.618 ± 0.054	1.0e-05	0.659 ± 0.044	0.565 ± 0.056	4.0e-10
THCA	0.746 ± 0.067	0.684 ± 0.068	1.1e-04	0.771 ± 0.065	0.752 ± 0.062	2.5e-01	0.727 ± 0.066	0.631 ± 0.084	3.1e-06
UCEC	0.667 ± 0.020	0.604 ± 0.025	3.5e-13	0.694 ± 0.046	0.644 ± 0.054	4.9e-05	0.678 ± 0.044	0.588 ± 0.055	6.1e-10

Table S2: **Comparison of c-indexs of Cox models trained with and without data pooling.**

Cancer Type	CLgCox-nnet	CLCox-nnet	p-value	CLgCox-XGB	CLCox-XGB	p-value	CLgCox-EN	CLCox-EN	p-value
BLCA	0.710 ± 0.040	0.681 ± 0.040	7.1e-03	0.704 ± 0.038	0.693 ± 0.037	1.9e-01	0.708 ± 0.043	0.692 ± 0.035	1.2e-01
CESC	0.776 ± 0.036	0.756 ± 0.037	9.9e-03	0.748 ± 0.053	0.761 ± 0.040	2.1e-01	0.770 ± 0.034	0.742 ± 0.058	7.8e-02
COAD	0.692 ± 0.033	0.677 ± 0.033	3.6e-02	0.719 ± 0.071	0.688 ± 0.048	1.4e-02	0.697 ± 0.038	0.671 ± 0.041	7.9e-03
GBM	0.675 ± 0.042	0.670 ± 0.048	6.4e-01	0.696 ± 0.049	0.690 ± 0.037	4.9e-01	0.680 ± 0.034	0.677 ± 0.035	2.8e-01
HNSC	0.687 ± 0.036	0.696 ± 0.058	6.2e-01	0.680 ± 0.031	0.682 ± 0.038	9.8e-01	0.689 ± 0.026	0.663 ± 0.028	5.5e-05
KIRC	0.793 ± 0.035	0.795 ± 0.038	7.4e-01	0.819 ± 0.047	0.787 ± 0.040	1.8e-03	0.780 ± 0.031	0.772 ± 0.031	2.9e-01
KIRP	0.879 ± 0.041	0.879 ± 0.033	5.9e-01	0.896 ± 0.054	0.894 ± 0.048	8.7e-01	0.879 ± 0.041	0.880 ± 0.039	8.0e-01
LGG	0.759 ± 0.027	0.746 ± 0.027	5.1e-02	0.805 ± 0.046	0.773 ± 0.039	1.7e-03	0.766 ± 0.033	0.758 ± 0.032	3.8e-01
LIHC	0.694 ± 0.033	0.680 ± 0.049	1.4e-01	0.695 ± 0.042	0.678 ± 0.034	3.5e-02	0.675 ± 0.027	0.658 ± 0.031	1.8e-02
LUAD	0.683 ± 0.021	0.678 ± 0.030	1.5e-01	0.685 ± 0.042	0.672 ± 0.036	1.9e-01	0.661 ± 0.030	0.657 ± 0.035	6.8e-01
LUSC(LC)	0.654 ± 0.052	0.619 ± 0.040	1.0e-03	0.676 ± 0.033	0.670 ± 0.040	2.6e-01	0.710 ± 0.034	0.661 ± 0.040	6.2e-07
LUSC(SM)	0.660 ± 0.047	0.619 ± 0.040	2.7e-04	0.660 ± 0.042	0.670 ± 0.040	3.3e-01	0.692 ± 0.034	0.661 ± 0.040	6.8e-04
SKCM	0.610 ± 0.023	0.601 ± 0.024	1.1e-01	0.634 ± 0.044	0.617 ± 0.028	8.3e-02	0.612 ± 0.023	0.602 ± 0.027	1.5e-01
STAD(DS)	0.641 ± 0.038	0.599 ± 0.034	5.1e-06	0.708 ± 0.056	0.676 ± 0.047	1.3e-02	0.689 ± 0.042	0.659 ± 0.044	2.5e-03
STAD(GG)	0.657 ± 0.028	0.599 ± 0.034	1.2e-10	0.709 ± 0.048	0.676 ± 0.047	3.3e-03	0.690 ± 0.040	0.659 ± 0.044	1.5e-03
